# A 3.55‐µm Ultrathin, Skin‐Like Mechanoresponsive, Compliant, and Seamless Ionic Conductive Electrode for Epidermal Electrophysiological Signal Acquisition and Human‐Machine Interaction

**DOI:** 10.1002/EXP.20240232

**Published:** 2025-07-08

**Authors:** Likun Zhang, Peiwu Qin, Huazhang Ying, Zhicheng Du, Chenying Lu, Minjiang Chen, Liyan Lei, Ziwu Song, Jiaju Chen, Xi Yuan, Canhui Yang, Vijay Pandey, Can Yang Zhang, Dongmei Yu, Peisheng He, Liwei Lin, Wenbo Ding, Xinhui Xing, Chenggang Yan, Jiansong Ji, Zhenglin Chen

**Affiliations:** ^1^ Shenzhen International Graduate School Tsinghua University Shenzhen China; ^2^ Tsinghua‐Berkeley Shenzhen Institute, Tsinghua Shenzhen International Graduate School Tsinghua University Shenzhen China; ^3^ Institute of Biopharmaceutical and Health Engineering, Shenzhen International Graduate School Tsinghua University Shenzhen Guangdong China; ^4^ Lishui Institute of Hangzhou Dianzi University Lishui Zhejiang Province China; ^5^ Department of Mechanical Engineering University of California Berkeley California USA; ^6^ Zhejiang Key Laboratory of Imaging and Interventional Medicine, Zhejiang Engineering Research Center of Interventional Medicine Engineering and Biotechnology The Fifth Affiliated Hospital of Wenzhou Medical University Lishui China; ^7^ School of Automation Hangzhou Dianzi University Hangzhou Zhejiang Province China; ^8^ Key Lab for Industrial Biocatalysis, Ministry of Education, Department of Chemical Engineering Tsinghua University Beijing China; ^9^ Soft Mechanics Lab, Department of Mechanics and Aerospace Engineering Southern University of Science and Technology Shenzhen China; ^10^ School of Mechanical, Electrical & Information Engineering Shandong University Weihai Shandong China

**Keywords:** skin‐like electrode, artifact‐free, ultrathin, gesture recognition, depression detection

## Abstract

Flexible ionic conductive electrodes, as a fundamental component for electrical signal transmission, play a crucial role in skin‐surface electronic devices. Developing a skin‐seamlessly electrode that can effectively capture long‐term, artifact‐free, and high‐quality electrophysiological signals remains a challenge. Herein, we report an ultra‐thin and dry electrode consisting of deep eutectic solvent (DES) and zwitterions (CEAB), which exhibit significantly lower reactance and noise in both static and dynamic monitoring compared to standard Ag/AgCl gel electrodes. Our electrodes have skin‐like mechanical properties (strain‐rigidity relationship and flexibility), outstanding adhesion, and high electrical conductivity. Consequently, they excel in consistently capturing high‐quality epidermal biopotential signals, such as the electrocardiogram (ECG), electromyogram (EMG), and electroencephalogram (EEG) signals. Furthermore, we demonstrate the promising potential of the electrodes in clinical applications by effectively distinguishing aberrant EEG signals associated with depressive patients. Meanwhile, through the integration of CEAB electrodes with digital processing and advanced algorithms, valid gesture control of artificial limbs based on EMG signals is achieved, highlighting its capacity to significantly enhance human‐machine interaction.

## Graphical abstract

The 3.55 µm ultrathin CEAB electrode is a flexible, self‐healing ionic gel with skin‐like mechanical properties, designed for stable, artifact‐free collection of electrophysiological signals during motion. This design ensures reliable long‐term monitoring for applications like depression detection via EEG, reflex arc detection, and hand gesture recognition through EMG, with potential for robotic hand control.

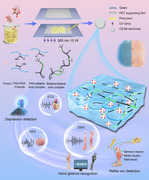

## 1 Introduction

Flexible ionic conductive electrodes play a pivotal role in skin‐surface electronic devices by facilitating electrical signal transmission [1]. They are indispensable for tasks such as continuous, non‐invasive health monitoring [2, 3, 4] and swift, efficient human‐computer interactions [5, 6, 7]. For effective long‐term acquisition of electrophysiological signals from the skin surface, electrode materials must possess specific attributes: adhesion [8], stretchability [9], Young's modulus similar to skin [9], excellent conductivity [10], and self‐healing [11] after external damage. Two types of skin electrodes: metal dry electrodes [12, 13] and hydrogel wet electrodes [14], are available in the market. While metal dry electrodes have stable physical and chemical characteristics, their limited stretchability and adhesive capabilities can cause signal distortions due to human movement [15]. In contrast, hydrogel electrodes excel in biocompatibility [16], conductivity, and skin conformity [17]. Yet, they lose water over time [18], diminishing their conductivity, stretchability, and softness; especially freezing at low temperatures and dehydration at high temperatures can compromise signal acquisition [19].

Deep eutectic solvents (DESs) are promising, safe, and stable alternatives to conventional soft ionic conductors [20]. Comprising hydrogen bond donors (e.g., ethylene glycol, EG) and acceptors (e.g., choline chloride, ChCl) [21], these solvents are water‐free, have low vapor pressure, and exhibit strong conductivity and biocompatibility [22, 23]. The composite advantages of DESs have spurred rapid development in ionic conductors [24]. Li et al. combined acrylic acid (AA) with DESs to prepare the DES gel with stretchability (>1000%), high conductivity (1.26 mS cm^−1^), and high adhesion to the skin (∼100 N m^−1^) [25]. Despite these advancements, creating a DES gel with both self‐healing and skin‐like mechanical properties remains a challenge. Human skin is self‐repairable, restoring both its mechanical and electrical functionalities [26]. Distinctively, human skin exhibits a nonlinear stress–strain relationship, resembling the shape of J [27]. While it feels soft upon touch, and swiftly stiffens under high strains to prevent damage. Inspired by skin's mechanic properties, a hybrid network that blends weakly complex zwitterions with robust hydrogen bond interactions can emulate skin's softness while rapidly stiffening under strain, mimicking its protective mechanism.

In this work, we develop an ionic, conductive, compliant, dry, adhesive, and self‐healing electrode for epidermal electrophysiology monitoring, depression detection, and human‐machine interaction. We use ChCl and EG as the deep eutectic solvent (DES), betaine as the zwitterionic network and crosslinker, and AA to form the conductive framework. This novel CEAB electrode surpasses traditional Ag/AgCl gel electrodes in epidermal biopotential monitoring. It ensures improved conductivity across a wide temperature range, offers gentle conformability to the skin, and significantly reduces noise levels during dynamic detections. We can use the CEAB electrodes to capture high‐quality epidermal biopotential signals consistently, such as electrocardiogram (ECG), electromyogram (EMG), and electroencephalogram (EEG) [28], even amidst movement. Furthermore, we develop a machine‐learning algorithm that effectively identifies abnormal EEG patterns in patients with depression. Meanwhile, by integrating this electrode with the shallow convolutional neural network (CNN) algorithm, we demonstrate prosthetic limb control as gesture replication based on EMG signals. A comparison of CEAB electrodes to representative electrodes for biopotential applications is shown in Table S1.

## 2 Results

### 2.1 Fabrication and Characterization of CEAB

**FIGURE 1:**
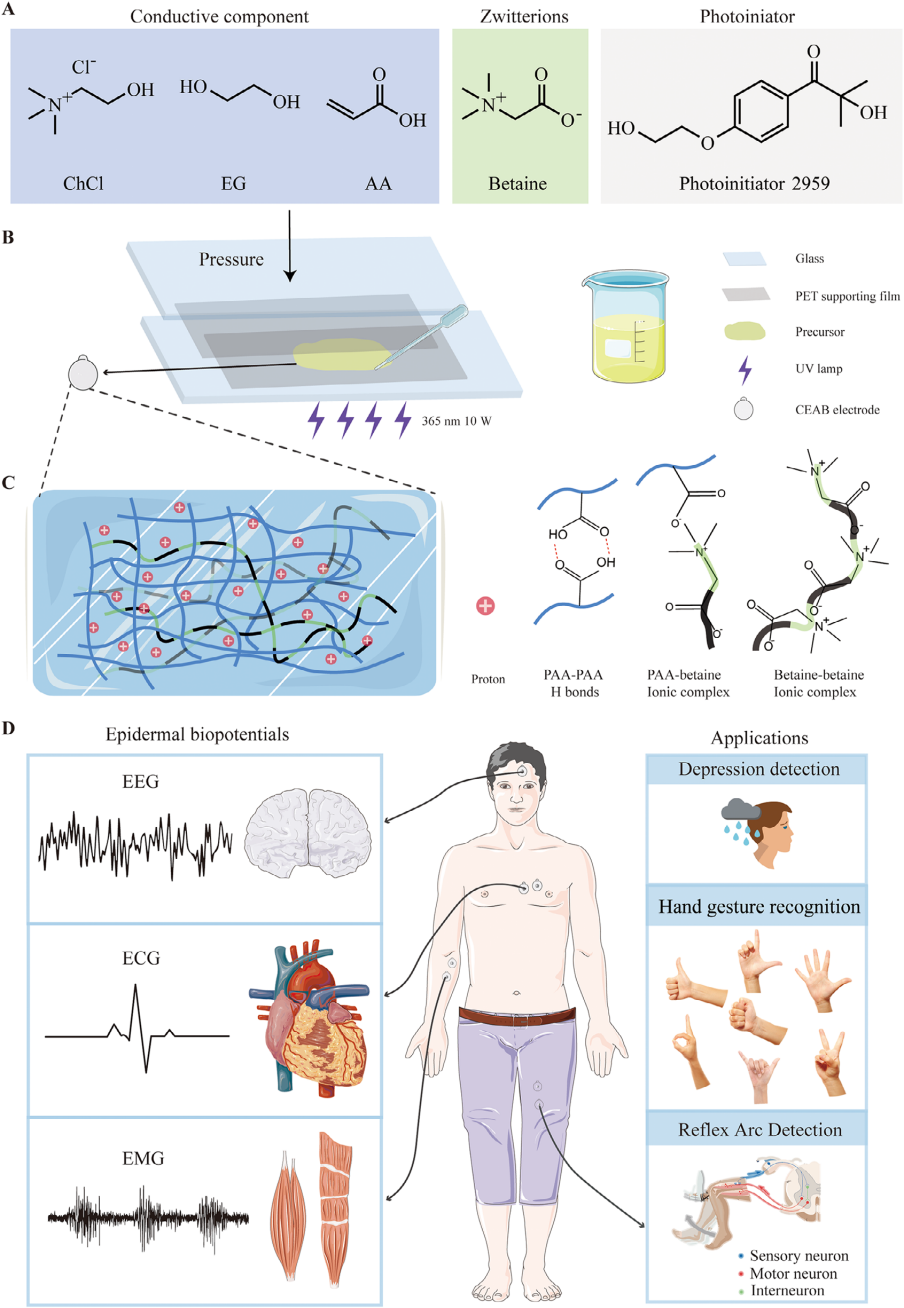
Schematic illustration for the preparation and application of CEAB films. (A) Chemical structure of ChCl, EG, AA, zwitterions, and photoinitiator. (B) Fabrication of the CEAB films: first, prepare the precursors; second, cast the precursor between the PET films and maintain pressure exertion; third, polymerize the precursor. (C) Schematic structure of CEAB elastomer. (D) CEAB gel for epidermal biopotentials’ detection and application.

We first develop a method to prepare CEAB film with controlled thickness and scalability, based on the pressure exertion. The fabrication process of the electrode is illustrated in Figure 1B (Further setup details in Figure S1A, Supporting Information). First, the DES is synthesized by mixing ChCl and EG, stirred at 80°C for 20 min. Then, monomer AA is dissolved in DES to form a clear solution [25]. After that, the betaine as zwitterion, and Irgacure 2959 as the photoinitiator are dissolved in the solution to prepare the CEAB precursor. The precursor solution consists of ChCl, EG, AA, and betaine in a molar ratio of 2:4:4:1, and the weight percentage of Irgacure 2959 is 0.1% (Figure 1A). The transparent precursor (Figure S1B) is then cast between two pieces of polyethylene terephthalate (PET) supporting films, covered by a flat glass mold, different forces are exerted on the surface to generate a precursor layer with different thicknesses. Upon polymerization of the precursor using UV light (365 nm, 10 W power, 5 min), the resulting CEAB gel (Figure 1C) serves as an adhesive and stretchable dry electrode for capturing epidermal biopotentials such as ECG, EMG, and EEG (Figure 1D).

**FIGURE 2:**
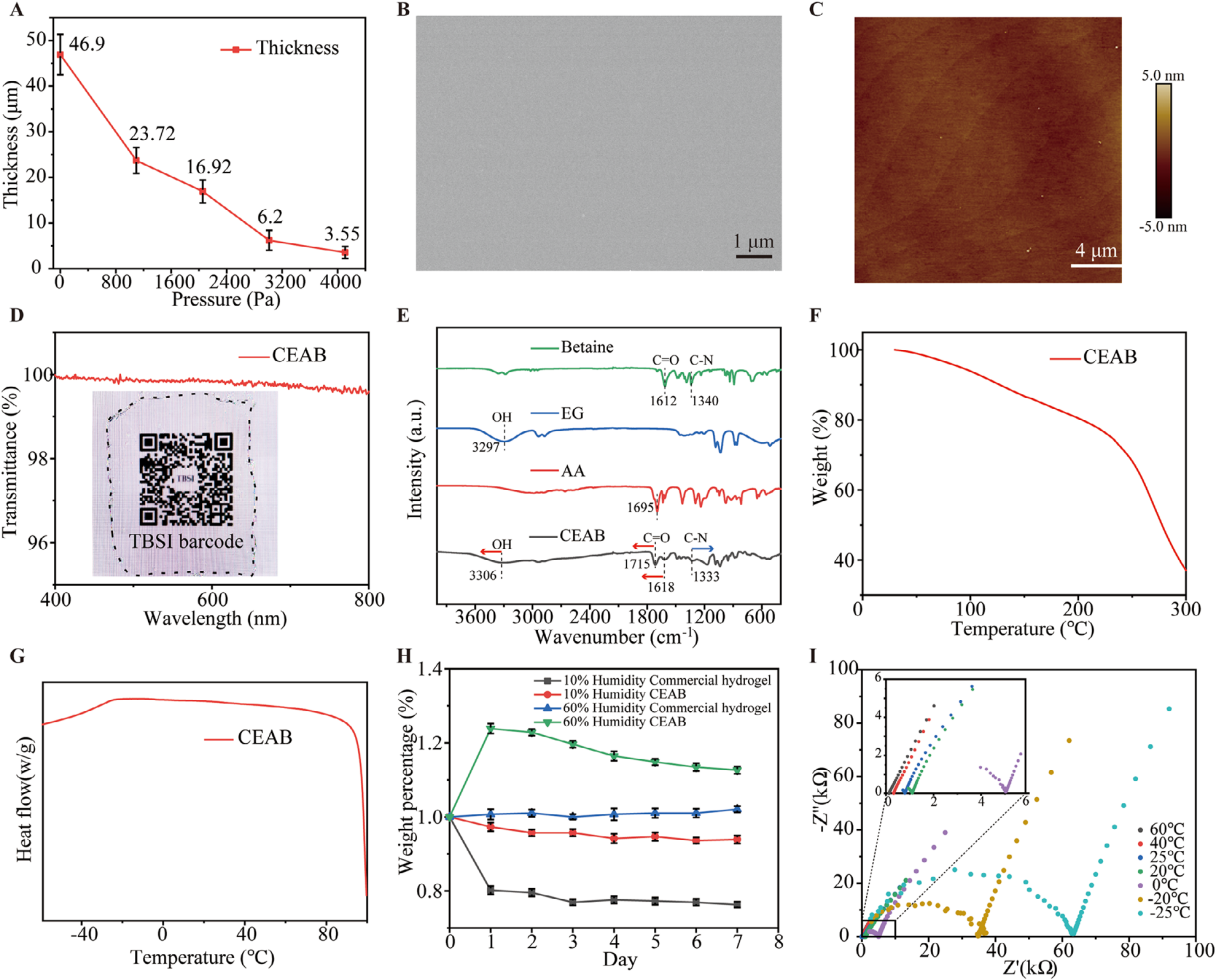
CEAB fabrication and characterization. (A) The relationship between thickness and pressure of CEAB film. CEAB film morphology characterized by (B) SEM (scale bar 1 µm) and (C) AFM (scale bar 4 µm). (D) Transmittance of CEAB film and photographs of the logo covered by CEAB films (inset). (E) ATR‐FTIR of individual components and CEAB film. (F) TGA curve of CEAB film. (G) DSC curve of CEAB film. (H) Weight changes of CEAB and commercial hydrogel at 10%, 60% RH within 7 days. (I) Demonstration of the electrical property of CEAB film: Nyquist plots at temperatures from −25°C∼60°C.

The thickness of the CEAB film is influenced primarily by two main factors: the magnitude and duration of pressure. Ensuring proper duration of pressure facilitates precursor diffusion between the PET films, subsequently impacting the film's thickness post‐photopolymerization. Precursor diffusion largely completes within 5 min under free load (Movie S1). Therefore, we set the pressure duration to 5 min. Figure 2A demonstrates that the CEAB film's thickness can be varied via the applied forces on the glass surface. Specifically, when controlling the pressure from 4109 to 0 Pa, the thickness is changed from 3.55 µm to approximately 46.9 µm. For films with a thickness surpassing 50 µm, we employ a molding strategy. This involves placing a spacer with a specific thickness between two glass plates and PET support films, followed by film removal post‐curing. We coat the PET film's surface with silicone oil to ease the CEAB film's separation, a critical step for maintaining the structural integrity of sub‐5 µm freestanding samples [29]. Scanning electron microscope (SEM) and atomic force microscope (AFM) (Figure 2B,C) both show the freestanding CEAB film's smooth morphology, indicating considerable macro‐scale homogeneity. Moreover, the AFM height image reveals that the CEAB film's surface roughness remains below 10 nm (Figure 2C and Figure S2). In comparison to traditional techniques, our pressure‐diffusion approach can provide a broad film thickness control range and extensive surface homogeneity. Figure S3 further illustrates the self‐adhesion of a 200 µm‐thick CEAB film on the palm, showcasing its capability to replicate complex, curved surfaces over large areas.

The CEAB film demonstrates an impressive optical transmittance exceeding 95% between 400–800 nm wavelengths (Figure 2D). This exceptional transmittance results from macroscopic averaging of compositional and structural factors. Furthermore, a QR code enveloped by this CEAB film remains effortlessly scannable, as depicted in the inset of Figure 2D. Utilizing attenuated total reflection‐Fourier transform infrared (ATR‐FTIR) spectroscopy, we delineate the characteristic peaks of the CEAB film and discern their interactions (Figure 2E). CEAB film and EG show OH peaks at 3307 and 3297 cm^−1^, this redshift of the OH group indicates the hydrogen bond formation [30], which is a characteristic of DES. For CEAB, both the *ν*(C═O) of AA and betaine shift to higher wavenumbers, while ν(C─N) of betaine shifts to lower wavenumbers, suggesting the formation of [N(CH_3_)_3_]^+^:[COO^−^] ion pairs [31]. Additionally, thermogravimetric analysis (TGA; Figure 2F) confirms the film's exceptional temperature stability, with minimal weight loss (<5%) until 82.5°C, and a decomposition temperature nearing 240°C, suitable for high‐demanding conditions. Concurrently, differential scanning calorimetry (DSC) in Figure 2G establishes that the CEAB's glass transition temperature (*T*_g_) remains below −60°C, underscoring its flexibility even at low temperatures. Figure S4 further demonstrates the flexibility of 2 mm thick CEAB film, that it can be easily twisted at both 25°C and −30°C. Figure 2H reveals that, in contrast to Ag/AgCl hydrogel, the CEAB gel exhibits a weight increase of approximately 15% at 60% relative humidity (RH) and experiences minimal weight loss (below 5%) at 10% RH over 7 days. This result underscores the CEAB film's potential for effective epidermal moisture retention. Figure 2I detail the temperature‐dependent ionic conductivity within a range of −50°C to 60°C. For temperatures above 20°C, the RH is controlled at 60%. To determine the electrical conductivity (*σ*) of CEAB film, the formula *σ* = *d*/*RS* is employed, where d represents the gel thickness, *S* signifies the gel area, and *R* denotes the value where the plot intersects the *Z*' axis. As temperature ascends, CEAB exhibits enhanced conductivity; for instance, its conductivity registers as 1.69 × 10^−2^, 1.33, and 8.18 mS·cm^−1^ at temperatures of −25°C, 25°C, and 60°C, respectively (Figure S5A, Supporting Information). The conductivity at 40% RH, 25°C is also measured as 1.06 mS·cm^−1^, compared with 1.33 mS·cm^−1^ at 60% RH, 25°C, with the increase of the RH, the conductivity increases by about 20.3%. Besides, the ionic conductivity's temperature dependency follows the Vogel–Fulcher–Tammann (VFT) relationship [32], demonstrating a robust congruence between theory and empirical findings (Figure S5B). Such elevated conductivities arise from rapid proton migration within the CEAB at high temperatures, inferred from its composition. DES contains ionized components such as hydrogen donors and hydrogen acceptors, which offer more protons to increase the conductivity. In addition, the low melting point of DES contributes to the free mobility of the protons, thus providing high conductivity of CEAB.

### 2.2 Mechanical Properties and Self‐Healing Ability

**FIGURE 3:**
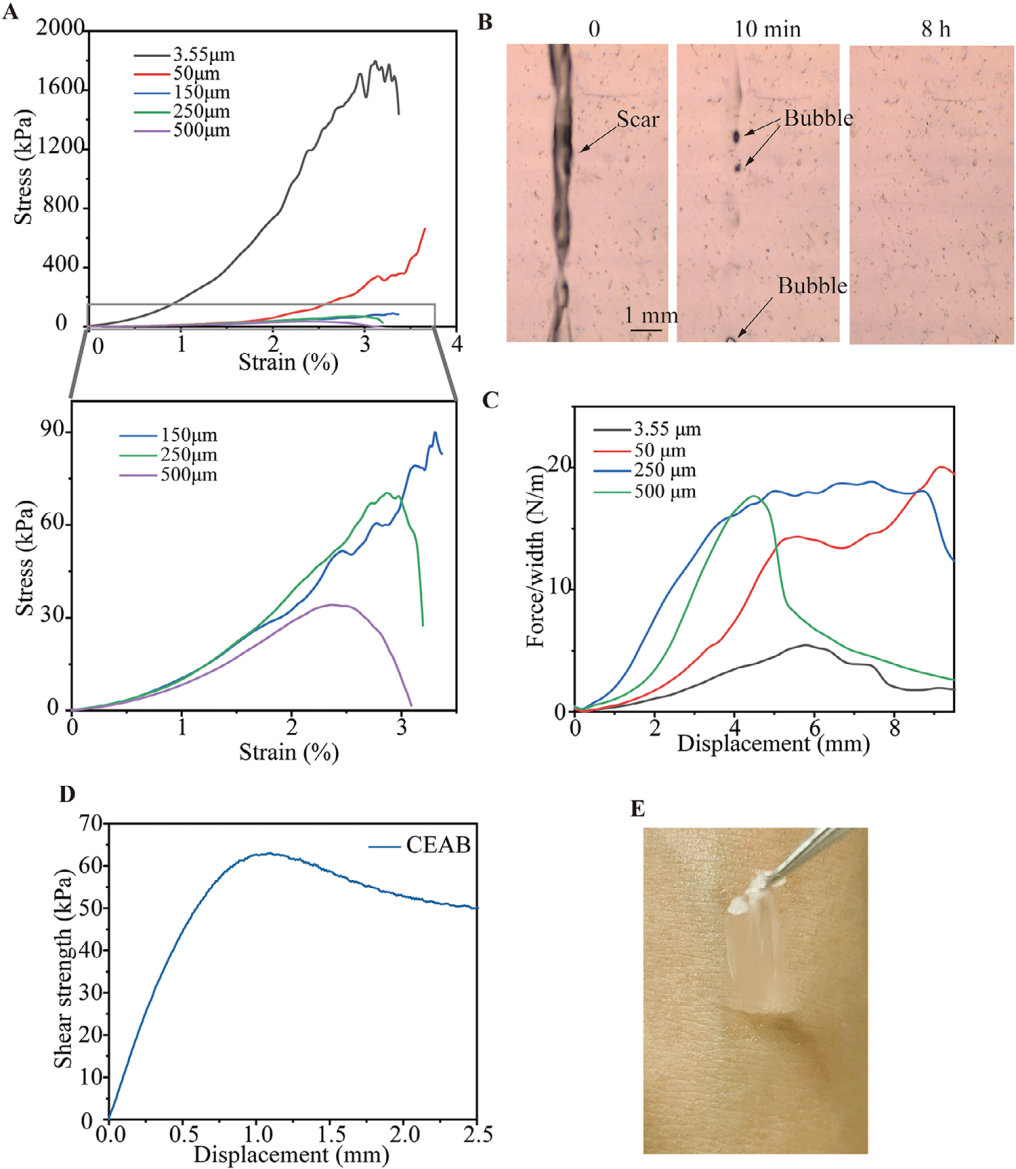
Mechanical properties and self‐healing ability of CEAB films. (A) Representative true tension stress‐strain curves of CEAB films with variable thicknesses, below is a zoomed‐in image of the data from 150–500 µm. (B) The bright‐field microscope images of a 2 mm thick CEAB film obtained at self‐healing intervals of 0, 10 min, and 8 h. (C) Representative curves of the peeling force per width (F/W) versus displacement with variable thicknesses of the CEAB films. (D) The representative curve of the shear strength versus displacement of a 500‐µm‐thick CEAB film. (E) A snapshot of the moment when the 500 µm thick CEAB film is peeled off from the dorsal hand.

Mechanical properties akin to the skin are pivotal for the application of epidermal electrodes, serving as a seamless interface between skin and electronics [9, 33]. To evaluate the mechanical attributes of the CEAB film, a uniaxial tensile setup is employed in 40% and 60% RH. At 60% RH, the true stress–strain curve (Figure 3A) exhibits strain‐stiffening behavior, resembling that of human skin, where the film initially feels soft and compliant but rapidly stiffens under increasing strain, enhancing its resistance to mechanical deformation. Notably, the true stress–strain response at 40% RH remains comparable (Figure S20), indicating that the CEAB film maintains consistent mechanical performance across different humidity conditions, making it suitable for diverse environmental settings. Additionally, Young's moduli of the CEAB films range from 3.23 to 59.74 kPa, aligning with values measured in the fibrous dermis (35–150 kPa) and the hypodermis (2 kPa) [34]. As delineated in Table S2, Young's modulus increases twentyfold as the film's thickness dwindles from 500 µm (3.23 kPa) to 3.55 µm (59.74 kPa). This enhancement stems from denser cross‐linking for thinner precursors under identical UV exposure durations. Impressively, the CEAB film can stretch approximately 800% (engineering strain) of its original length without exhibiting discernible mechanical failure, a capability aligning well with on‐skin electronics requirements, given that skin typically endures a maximum strain of around 30% [34].

CEAB gel demonstrates rapid self‐healing attributes, which meet the requirements of an ideal epidermal electrode that self‐recovers from external mechanical damage. As depicted in Figure 3B, the scar on the CEAB film vanishes entirely within 10 min at room temperature, with bubbles near the scar dissipating within 8 h (Movie S2). This self‐healing process is attributed to the electrostatic interaction in betaine, reversible H‐bonds in ChCl, EG, and polyacrylic acid, facilitating polymer chain diffusion at the interfaces. To assess the CEAB film's self‐adhesion on porcine skin, a 90° peeling experiment is conducted, as illustrated in Figure S15. Within a thickness range from 3.55 to 500 µm, the CEAB exhibits a peeling interface toughness ranging between 5–20 J m^−2^ (Figure 3C), demonstrating strong adhesive capability for artifact‐resistant epidermal electrical signal collection during movement. Figure S6A–D further verifies the strong adhesion to plastic tubes, paper, PET film, and rubber gloves, respectively. Concurrently, the 500 µm CEAB film demonstrates a shear strength of 63 kPa on the porcine skin (Figure 3D), translating to a maximal shear force of 12.60 N—70 times the 90° peel force (0.18 N). Despite its superior adhesive qualities, the CEAB film allows for effortless and comfortable removal, as depicted in Figure 3E. Unlike conventional tapes (3 M VHB) that often induce discomfort due to excessive adhesion and residual islands, the CEAB film ensures a user‐friendly detachment.

### 2.3 Conformability and Biocompatibility

CEAB films demonstrate remarkable conformability across diverse rough surfaces. Two primary factors influencing a material's conformability are its Young's modulus and thickness; a decrease in both parameters enhances the material's ability to conform to irregular surfaces [35]. Figure S7A–D reveals that both 3.55 and 200 µm CEAB films adhere tightly to human skin due to their ultrasoft nature, fostered by hydrogen bonding on both the electrode‐skin boundary and internal electrode, as well as weak electrostatic interactions in the electrode. And the process of transferring CEAB film to different substrates is shown in Figure S17. These CEAB films readily accommodate skin deformations, such as stretching and squeezing. In Figure S8A–C, a 3.55 µm CEAB film affixed to the dorsal hand synchronizes with skin movements seamlessly, elongating upon stretching and forming wrinkles similar to skin creases during compression. In contrast, PET adhesive tape partially loses contact with the skin, failing to emulate the skin's subtle wrinkles due to modulus disparities between the skin and the PET film. Additionally, the 3.55 µm CEAB film exhibits conformal adhesion to fruit peels, including apples and avocados, as depicted in Figure S9, whereas the PET tape demonstrates partial attachment from these fruit surfaces.

**FIGURE 4:**
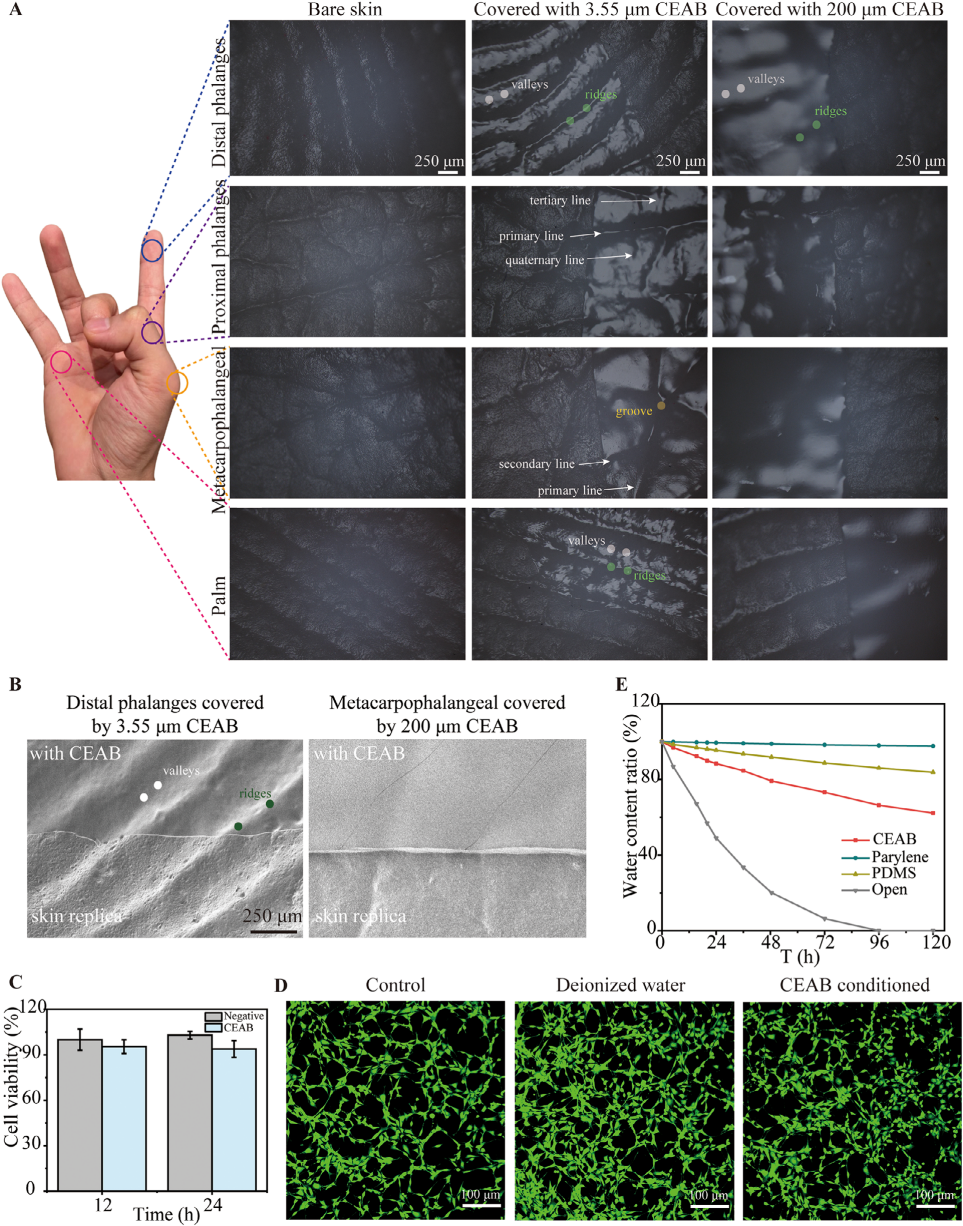
Conformability and biocompatibility of CEAB films. (A) The conformability of CEAB films with different thicknesses on the skin: The structure of a human hand (left) and optical images (right) depicting the surface texture of the bare replica and the replica covered by 3.55 and 200 µm CEAB film, respectively. (B) The SEM image of the replica covered by 3.55 and 200 µm CEAB film, respectively. (C) CCK‐8 results, and (D) confocal microscope images of NIH3T3 cells cultured with regular medium, deionized water, or regular medium plus CEAB extracts at 24 h, respectively. (E) The comparison of water vapor transmission rate for a 3.55 µm thick CEAB film, a 50 µm thick PDMS, and a 4 µm thick PET film at RH 40% for 5 days.

Besides, since the geometry of the glyphic patterns at hands varies at the different locations, 3.55 and 200 µm thick CEAB film is attached to different regions of the hand to investigate the local texture conformability, including distal phalanges, proximal phalanges, metacarpophalangeal, and palm. Silicon rubber is used as the human hand replica, and bare skin without an attached electrode is captured to demonstrate the primitive morphology. As depicted in Figure 4A, with a 3.55 µm thick CEAB film covering the surface, glyphic lines on proximal phalanges and metacarpophalangeal, including primary, secondary, tertiary, and even quaternary lines, can be distinguished under the optical microscope. Additionally, the 3.55 µm thick CEAB film precisely conforms to the ridges and valleys, revealing fine structures including dense and directionally varying grooves, as well as irregular elevations between furrows with high resolution. The intimate contact between the fingerprint replica and 3.55 µm CEAB film is further investigated using SEM (Figure 4B), which reveals a secure adherence of the 3.55 µm CEAB film to the fingerprint replica, indicating distinct ridges and valleys with no detectable formation of air gaps. However, as the thickness of the CEAB film increases to 200 µm, the ability to discern fine structures such as grooves and papules is restricted due to a size mismatch; specifically, the depth between ridges and valleys is typically less than 60 µm [36]. Nonetheless, covered by the 200 µm CEAB film, the ridges, and valleys on the distal phalanges and palm remain discernible due to their ultrasoft mechanical properties. In agreement with optical images, ridges, and valleys can be discerned on the metacarpophalangeal replica covered by 200 µm CEAB film, with no observable air gaps due to the ultrasoft mechanical properties.

The cytotoxicity assay with NIH3T3 cells confirmed the biocompatibility of the CEAB gel (Figure 4C). A further cytotoxicity assessment was conducted using live‐dead staining after 24 h of incubation with DMEM, deionized water, and CEAB‐conditioned DMEM. Fluorescence microscopy images revealed no significant decrease in cell viability in the CEAB extract hydrogel medium culture compared to the control group. The results suggest that CEAB exhibits excellent biocompatibility and holds promise for applications in biocompatible wearable electronic devices. The water vapor transmission ability of CEAB film is compared with common interactive materials for human‐machine interface substrates, such as PDMS and Parylene. After 5 days, CEAB had nearly 50% water vapor transmission, while PDMS and PET only had 10% or less water vapor transmission (Figure 4E). This indicates that CEAB film, as a wearable electrode material, will not block the skin surface and affect skin respiration. We summarize the overall comparison of our CEAB film with the most representative ionic gels in Table S3.

### 2.4 Electrode/Skin Contact Impedance and Epidermal Biopotential Detection

**FIGURE 5:**
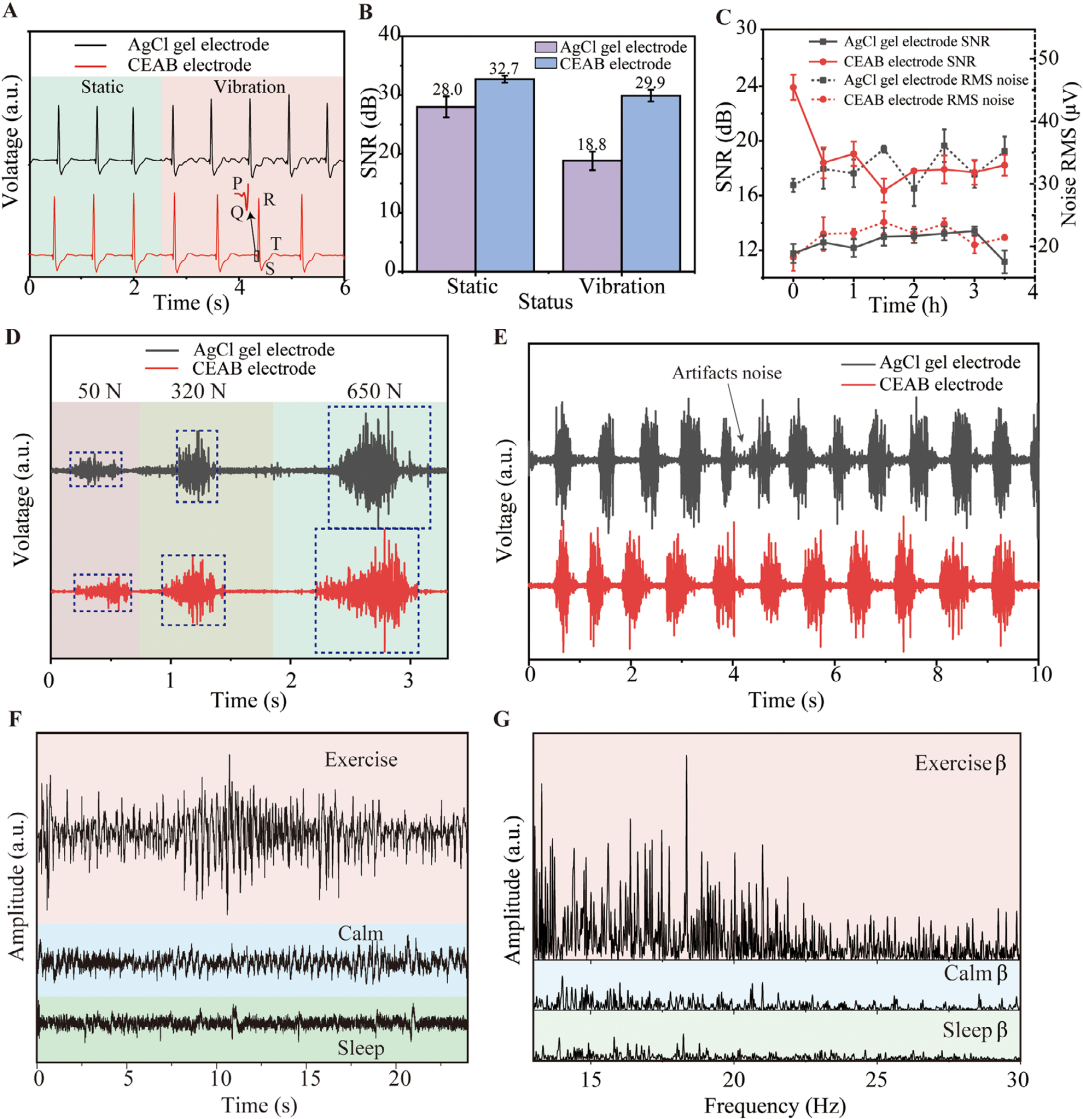
Biopotentials acquisition based on CEAB electrodes and signals quality evaluation. (A) ECG measured by Ag/AgCl (black) gel, CEAB electrode (red) without and with vibration. Rhythmics‐relevant P, Q, R, S, and T waves are identified, even when vibration is exerted near the CEAB electrode. (B) The SNR of the ECG signal collected by Ag/AgCl (purple) gel and CEAB (blue) electrode at static and vibration state, respectively. (C) The EMG SNR (full lines) and noise RMS (dotted lines) of Ag/AgCl (black) gel, CEAB electrode (red). (D) The EMG signals recorded using Ag/AgCl (black) gel and CEAB electrode (red) during grip force exertions of 50, 320, and 650 N, respectively. (E) The facial EMG signals were recorded by Ag/AgCl (black) gel and CEAB electrode (red) during smiling episodes. (F) The original EEG signals were recorded by CEAB electrode while (G) the β band EEG signals obtained through FFT of the original EEG signals, recorded during the volunteer's engagement in exercise, calmness, and sleep.

The electrode/skin contact impedance within 1 h of the CEAB electrode is measured in the frequency range of 1–10^4 ^Hz. The CEAB electrode shows stable electrode/skin contact impedance (Figure S10A,B). The average electrode/skin impedance with 1 h of 3.55 µm CEAB electrode is much lower than the 1.5 mm CEAB electrode and commercial Ag/AgCl gel electrode, that is, 9.83, 66.93, and 288.07 kΩ for the former at 1000, 100, and 10 Hz, respectively (Figure S10C–E). Stable and lower electrode/skin contact impedance contributes to high‐quality biopotential acquisition. Considering the ultrasoft mechanical properties, the adhesion to the skin, lower and stable electrode/skin contact impedance, and the self‐healing ability, we employ the 3.55 µm CEAB film for epidermal ECG, EMG, and EEG acquisition. The electrical connection interface between sensors and CEAB film is shown in Figure S18A–G. Figure 1D illustrates two CEAB electrodes attached to a volunteer's chest for ECG signal recording. Notably, rhythm‐related parameters—including P, Q, R, S, and T waves—are distinctly identifiable in both static and dynamic states, crucial for clinical diagnoses [37], as shown in Figure 5A. In contrast, the Ag/AgCl gel electrode exhibits higher noise levels and unstable peaks during motion. Benefitting from its robust adhesion and conformability, the CEAB electrode has superior signal‐to‐noise ratios (SNR) of 32.7 and 29.9 dB in static and dynamic states, respectively, surpassing the 28.0 and 18.8 dB of the Ag/AgCl gel electrode, as depicted in Figure 5B. This performance underscores the CEAB electrode's resilience against motion artifacts. As shown in Figure S16, the ECG signals recorded by CEAB electrodes are stable after repeated use on the skin ten times. High retention of adhesion and contact impedance is responsible for reliable reusability.

Additionally, to capture muscle biopotentials, two CEAB electrodes are affixed to the forearm as working electrodes, while one is placed on the elbow as a reference electrode. By sustaining a grip force of 50 N, we assess the SNR and noise level of CEAB electrodes during EMG signal acquisition. Figure 5C illustrates the noise analysis using RMS (root mean square) on baseline signals acquired during rest periods. The average noise level of the CEAB electrode (21.7 µV) is reduced by 33.6% over the 3.5 h monitoring period compared to the Ag/AgCl gel electrode (32.7 µV). Furthermore, the CEAB electrodes consistently outperform Ag/AgCl gel electrodes in SNR, averaging 12.5 dB—a 48.9% improvement over the 18.7 dB of the Ag/AgCl gel electrodes. This evidence solidifies that CEAB electrodes offer superior noise reduction and enhanced SNR compared to commercially available Ag/AgCl gel electrodes. Subsequent tests involving varied grip forces of 50, 320, and 650 N confirm that EMG signals remain distinguishable, aligning with the Ag/AgCl gel electrodes (Figure 5D). Notably, the CEAB electrode captures more stable EMG signals with minimal baseline fluctuations, demonstrating superior reliability. Figure S11 further illustrates that CEAB electrodes effectively differentiate EMG signals across various hand gestures, essential for human‐machine interface applications. Coincidentally, Movie S4 further demonstrates the EMG signals of the forearm during various single‐finger and hand movements.

The facial muscles typically exhibit non‐uniform distribution, and facial skin is prone to wrinkles development with facial expressions, undergoing substantial deformation [38]. Acquiring stable and high‐quality facial EMG signals using flexible electrodes with excellent conformality remains a significant challenge. To ascertain the efficacy of CEAB electrodes as proficient facial EMG electrodes, we collect facial EMG data during volunteers' smiling episodes, with Ag/AgCl gel electrodes serving as a reference. As illustrated in Figure 5E, both electrodes demonstrate the capability to capture signals of good quality during volunteer smiles. However, the CEAB electrodes exhibit lower baseline noise. Throughout the entire data‐acquisition process, the signals acquired by the CEAB electrodes have consistent baseline noise levels. In contrast, with an increasing time of smiles, substantial noise emerges between adjacent peaks for the Ag/AgCl gel electrode after 4 s. This occurrence can be attributed to the wrinkles induced by smiling, leading to motion artifacts between the Ag/AgCl gel electrode and the skin surface. The superior stretching ability and Young's modulus comparable to that of the skin of CEAB film facilitate accurate EMG signal acquisition, particularly during facial motion.

Given the CEAB electrode's superior resistance to motion artifacts, minimal baseline noise, and enhanced SNR compared to Ag/AgCl gel electrodes, we utilize it for long‐term EEG recordings on a volunteer, spanning up to 12 h. Throughout this long duration, the volunteer is engaged in varied activities, including sleep, exercise, and relaxation. Notably, the quality of EEG signals remains consistent, even when the volunteer perspires during physical activities. Figure 5F illustrates three distinct EEG signals corresponding to different mental states: heightened activity during exercise and reduced activity during sleep. By employing fast‐Fourier transformation (FFT), we segment the EEG brainwaves into specific frequency bands: *δ* (0–2.5 Hz), *θ* (3.5–6.75 Hz), *α* (7.5–11.75 Hz), *β* (13–30 Hz), and *γ* (31–50 Hz) [39]. The *β* waves are associated with increased energy levels and can reflect the degree of mental concentration and physical involvement [40, 41]. Then we extract the *β* wave from the signal and compare the discrepancy. As depicted in Figure 5G, the intensity of *β* wave during the exercise state is much stronger than that at rest or sleep status, which accords with the fact that *β* usually increases during physical exertion.

### 2.5 Clinical Detection and Depression Detection

**FIGURE 6:**
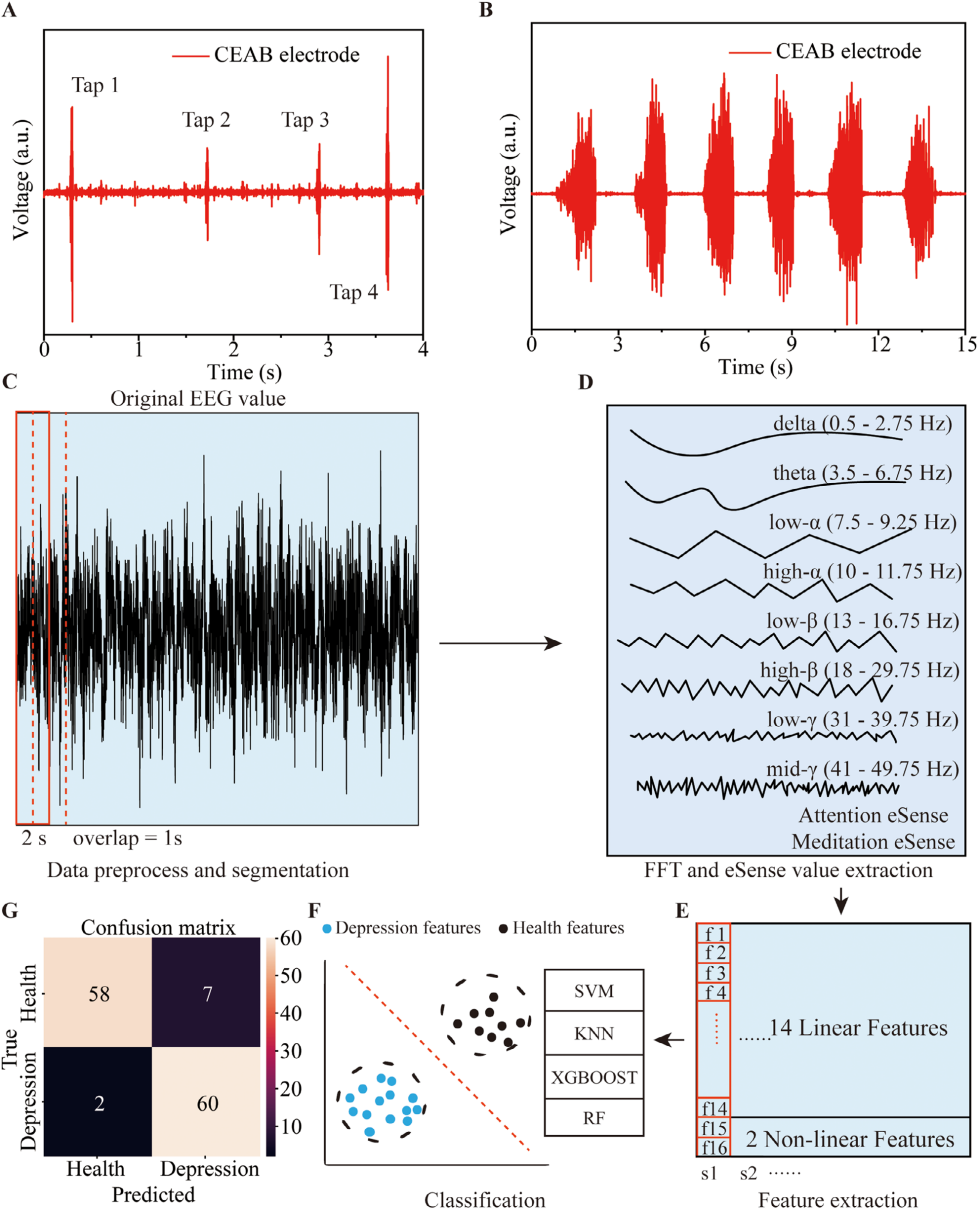
Clinical detection and depression detection based on CEAB electrodes. (A) The knee jerk reflex EMG signals collected by CEAB electrodes during four hammer taps. (B) The thigh muscles' EMG signals are collected by CEAB electrodes during leg voluntary contraction. (C) The EEG signal preprocesses and sample segmentation. (D) Eight frequency bands are acquired after the FFT and eSense values are extracted. (E) The 16 features are extracted, with 14 linear and 2 non‐linear included. (F) The feature data are fed into machine learning models for training and classification. (G) Confusion matrix of depression prediction by RF model on test sets, with true categories along the row and predicted types along the column.

The knee jerk reflex is classified as a monosynaptic stretch reflex. In clinical settings [42], tendon reflex examinations are commonly employed to assess the circuit integrity of the stretch reflex arc and to evaluate motor neuron functionality. To diagnose spinal pathway function, we affix CEAB electrodes to a healthy volunteer's thigh muscle. Consistent with hospital tests, when the kneecap is tapped with a small hammer, the CEAB electrodes capture a rapid surge in muscle electrophysiological activity, as depicted in Figure 6A. This observation aligns with findings from clinical studies and literature. Moreover, the CEAB electrodes monitor voluntary thigh muscle contractions during leg extensions. Unlike the knee‐jerk reflex, these stronger contractions manifest larger amplitudes and prolonged durations due to the engagement of multiple motor units, resulting in more pronounced motor unit action potentials, as illustrated in Figure 6B.

Depression is a severe mental disorder characterized by persistent feelings of sadness and potential suicidal tendencies [43, 44]. According to the World Health Organization (WHO), as of 2023, over 264 million people globally are afflicted with depression, underscoring the urgency for early diagnosis and intervention [45]. Given EEG's ability to provide a direct reflection of brain neurological activity with high temporal resolution, EEG is increasingly recognized as a potent tool for non‐invasive depression diagnosis [46, 47]. Utilizing the CEAB electrode, we have designed a machine‐learning approach to analyze single‐channel EEG data of subjects at rest state, facilitating swift and reliable depression diagnosis, as depicted in Figure 6C–F. Specifically, real‐time data collection is conducted, followed by preprocessing with Python 3.8. EEG signals inherently display temporal variability and dynamics, and different frequency components correspond to different temporal and physiological states [48]. To build extensive training datasets for machine learning, we segment the EEG data into 2‐s intervals, incorporating a 50% overlap to capture the intricate and dynamic attributes of EEG effectively (Figure 6C). This windowing strategy has been demonstrated to encapsulate relevant information across varied EEG types, thereby enhancing classification outcomes [49]. Our dataset encompasses 633 samples, balanced between healthy and depression‐afflicted samples. As showcased in Figure 6D, we apply FFT to the original EEG signals, delineating frequency bands and deriving eSense values via NeuroSky's algorithm [50, 51]. This algorithm can express the mental state information (attention and meditation) of the human brain with eSense values. Then we extract two distinct feature categories from the EEG data: linear attributes, including variance, absolute power, mean, and coherence, juxtaposed against nonlinear attributes like entropy and C0‐complexity [52] (Figure 6E). Linear features contain signal specificity, whereas nonlinear attributes underscore intricacy and stability [52]. Subsequently, these features are concatenated and then input into a range of classifiers, namely supporting vector machine (SVM) [53], K‐nearest neighbor (KNN) [54], XGBoost [55], and random Forest (RF) [56] for depression detection (Figure 6F). As illustrated in Table 1, the RF performs the best accuracy of 92.91%, recall of 92.91%, and precision of 93.19% on the test dataset. Figure 6G further illustrates the confusion matrix of RF models on depression prediction.

**TABLE 1:** Machine learning model results.

	KNN	XGBoost	SVM	RF
Precision	89.78%	89.78%	69.28%	93.19%
Recall	89.76%	89.76%	69.29%	92.91%
F1 score	89.77%	89.77%	69.28%	92.91%

To delve deeper into understanding feature contributions for predicting depression, we employed the Gini importance method within scikit‐learn for enhanced feature analysis [57]. Utilizing the RF model, individual feature importance is computed and graphically depicted in Figure S12. The histogram highlights four paramount features: mean attention, standard deviation σ, mean of the low *γ* band, and mean of the *δ* band (Figure S13A–D). Approximately 80% of individuals with depression exhibit diminished attention compared to their healthy counterparts. Furthermore, ∼20% of samples manifest higher *σ*, where higher variance indicates greater emotion change among subjects with depression [58]. Notably, the low *γ* band's sensitivity to emotional nuances indicates a trend that a majority of depression patients register elevated low *γ* values, thereby identifying these metrics as potential depression biomarkers [59, 60]. Additionally, over 80% of depression samples have lower mean *δ* values, which is correlated to heightened psychological distress, potentially amplifying depression vulnerability [61]. Leveraging CEAB electrodes, we build a single‐channel RF model finding digital depression biomarkers. EEG with CEAB electrodes offers an efficient, user‐friendly, and feasible solution for depression detection via a single‐channel wearable EEG headband.

### 2.6 Hand Gesture Replication by Robotic Hands

Hand gestures play a pivotal role in conveying concise messages and carrying emotional implications, making them essential in both realistic and digital communication, especially within human‐machine interaction applications [62]. While traditional methods of gesture sensing and recognition primarily depend on algorithms to semantically interpret images or videos, they often face challenges due to environmental interferences such as obstructed objects and varying lighting conditions [63]. Unlike traditional methods, EMG, which captures signals before muscle contraction, provides a robust solution for hand gesture recognition without interference from external environmental factors [64]. We utilize CEAB electrodes to record forearm EMG signals from volunteers performing six distinct hand gestures. The captured signals are subsequently combined with sophisticated algorithms and embedded techniques to instruct robotic arms to mimic the recorded hand movements. The electrical connection and setup for real‐time recording EMG signals are demonstrated in Figure S19.

**FIGURE 7:**
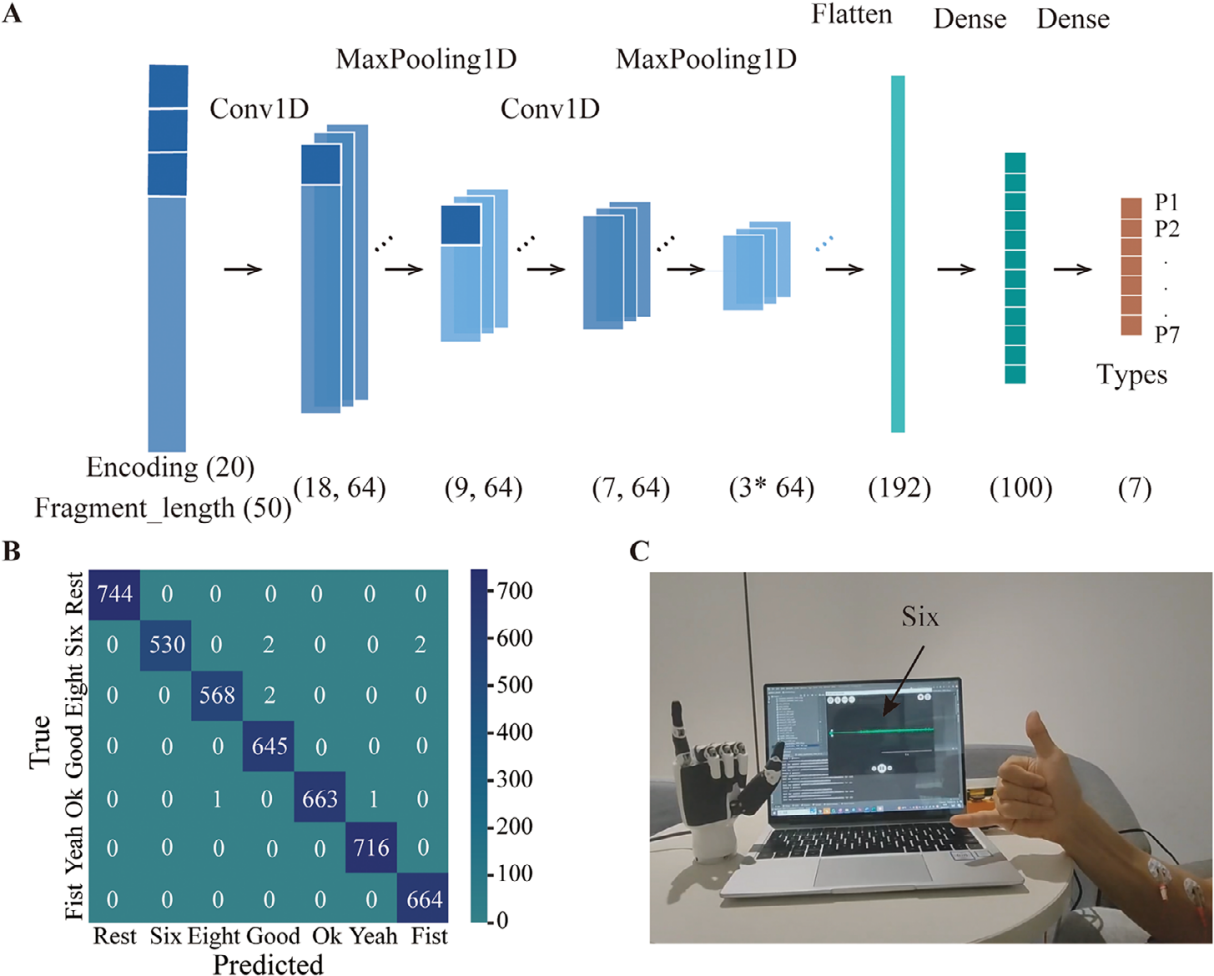
Gesture recognition and gesture replication by robotic hands. (A) Architecture of the gesture‐recognition CNN model. (B) Confusion matrix of gesture recognition, with actual gesture type along the rows and predicted gesture type along the columns. (C) By combining the signal acquisition with CEAB electrodes and data analysis with the CNN model, the EMG biopotentials can instruct the robotic arm to perform the gesture of the number “six”.

First, we collect an EMG dataset of different gestures from volunteers, encompassing categories of “rest,” “six,” “eight,” “good,” “yeah,” “ok,” and “fist”. These samples are carefully segmented into intervals with 1000 data points with a 95% overlap, strategically designed to augment the dataset and encapsulate essential biopotential information related to gesture motion. Subsequently, we establish a two‐layer convolutional neural network (CNN) model tailored for efficient gesture classification. Figure 7A provides a visual representation of this CNN model's architecture. The analysis begins by reshaping the input data into a 2D array, followed by convolutional and pooling layers to extract pertinent features. The following steps include flattening and deploying dense layers to achieve multi‐label classification. Figure 7B presents the confusion matrix results for gesture recognition, where the predicted and actual type counts are consistent, differing only in one or two instances. The training loss and accuracy are shown in Figure S14, highlighting the model's gesture recognition capabilities. Our results achieve 99.78% for precision, recall, and accuracy due to larger signal differences (Figure S11B) captured by CEAB electrodes among various gestures.

Subsequently, we utilize the algorithm to recognize gestures and instruct the robotic arms to replicate human gestures. Upon feeding the forearm biopotential data of various gestures into the trained models, the system returns a predicted label in approximately 150 ms. This label is then relayed to the robotic arm, instructing it to execute the corresponding gesture. To account for the time delay required for the robotic arm's movement, we set an appropriate delay before accepting the subsequent EMG input. Figure 7C demonstrates the hand gesture “six” replication by the robotic arm. Details of the forearm biopotential driving the robotic arm to imitate human gestures can be found in Movie S5.

## 3 Conclusion and Discussion

In conclusion, our study develops an ionic, flexible, dry, and self‐healing electrode, named “CEAB,” through the utilization of deep eutectic solvents (DESs) and ionically cooperative conductors. We propose a streamlined method for crafting an ultrathin ionic electrode film, achieving a thickness of as small as 3.55 µm. The CEAB electrode has exceptional attributes, including high conductivity, minimized noise levels, and the uninterrupted capture of epidermal biopotential signals (e.g., ECG, sEMG, EEG) during dynamic detection. Through seamless integration with digital signal processing and analysis algorithms, using a one‐channel wearable device, the electrode adeptly identifies abnormal EEG signals associated with depressive patients in clinical scenarios. Furthermore, this integration enables hand gesture repetition by robotic arms based on EMG signals, underscoring the transformative potential of this technology in health monitoring and human‐machine interactions.

## 4 Experimental Methods

### 4.1 Materials

Choline chloride (ChCl) (AR, 98%), ethylene glycol (AR ≥ 99%), acrylic acid (AA) (AR ≥ 99%), betaine (AR, 98%), Irgacure 2959 (AR, 98%), were all purchased Shanghai Macklin Biochemical Company (China). All reagents are used without further purification.

### 4.2 Preparation of CEAB Film

To prepare the deep eutectic solvent (DES), we first mixed the hydrogen bond acceptor, choline chloride (ChCl), and the hydrogen bond donor, EG, in a 1:2 molar ratio. Following this, the mixture was stirred at 100°C for 2 h. Then, acrylic acid (AA) was dissolved in the DES. Subsequently, we added betaine and Irgacure 2959 to the solution and vigorously stirred the mixture for 1 h until it dissolved completely, resulting in a clear precursor solution. The precursor solution consisted of ChCl, EG, AA, and betaine in a molar ratio of 2:4:4:1, and the weight percentage of 2959 was 0.1%. Afterward, the solution was allowed to stand overnight to eliminate all bubbles. Next, we coated the CEAB precursor between two PET release membranes pre‐treated with silicone oil and affixed it to the surface of a flat glass mold. Afterward, another flat glass was positioned above. Uniform pressure was applied to the surface glass, and the duration was set as 5 min. The thickness of the precursor layer could be controlled by adjusting the pressure and duration applied to the glass surface, for thickness more than 50 µm, a spacer of specific thickness is put between the PET films. The glass mold was placed 5 cm above a 365‐nanometer UV lamp with an output of 10 W and exposure of 5 min. After photopolymerization, an intact CEAB film is formed between two supporting PET films. Then we used the scissors to obtain a CEAB film of specific sizes and shapes. To prepare CEAB film of more complex shapes, the laser cutter is employed for its patterned, designable advantages.

### 4.3 Scanning Electron Microscope (SEM)

The gross morphology of a freeze‐dried CEAB film was observed via the Hitachi SU8010 under an accelerating voltage of 5 kV. The gel samples were then freeze‐dried for 24 h and sputter‐coated with gold before imaging. For the gel/skin replica samples, a 3.55 and 200 µm gel film was cut into small pieces with sharp edges and then directly attached to an EcoflexTM rubber skin replica. To remove the remaining organic solvent, all samples were freeze‐dried before being introduced to the SEM chamber.

### 4.4 Fourier Transform Infrared Spectroscopy (FTIR)

The samples were dried in a vacuum drying oven before testing. FTIR spectra of the samples were recorded using a spectrometer (Thermo Scientific Nicolet iS50) within the wavenumber range of 400 to 4000 cm^−1^.

### 4.5 Thermogravimetric Analysis (TGA)

After drying the gels, thermodynamic properties were analyzed using a thermogravimetric analyzer (NETZSCH's STA 449 F3 Jupiter). The initial mass of the sample was 5–10 mg, and it was heated from room temperature to 600°C at a rate of 10°C/min under a nitrogen (N_2_) atmosphere.

### 4.6 Differential Scanning Calorimeter (DSC)

The thermal transition behavior of the polymers was investigated using differential scanning calorimetry (Mettler‐Toledo DSC 3). The initial mass of the sample was 20–50 mg, and heating was performed at a rate of 10°C/min within the temperature range of −60 to 100°C under a nitrogen (N_2_) atmosphere.

### 4.7 The Freezing Stretch Tests

Ag/AgCl gel and CEAB samples (6 cm in length, 1 cm in width, and 200 µm in thickness) were exposed to −30°C for a duration of 24 h. Following this cold exposure, the samples were twisted to assess their flexibility.

### 4.8 The Moisture Retention Tests

CEAB and Ag/AgCl gels with identical weight and shape were kept at consistent temperatures of 37°C while exposing them to varying RH levels (RH: 10 % and 60 %) for a duration of 7 days. The weight of the samples was recorded at different time intervals during the test. Weight loss was calculated using the following formula:

$$\mathrm{Weight}\;\mathrm{percent}\;\left(\%\right)=W_{t}/W_{0}*100\;\%$$

Here, $W_{0}$ represents the initial weight of the gel and $W_{t}$ is the weight after storage at a specific time.

### 4.9 Electrical Measurement

To determine the ionic conductivities of the materials, we performed complex impedance measurements using an electrochemical workstation (PARSTAT 4000A, Princeton) across a frequency range from 0.1 Hz to 1 MHz, employing an alternating‐current sine wave with an amplitude of 500 mV. In this procedure, a CEAB sample was cut into a cylindrical shape with dimensions of 6 mm in diameter and 3 mm in depth, and it was placed between two round steel electrodes. The material was kept at the specific temperature of −25°C, −20°C, 0°C, 20°C, 25°C, 40°C, and 60°C for 30 min before testing.

To obtain the bulk resistance (*R*) of the materials, we identified the intercept on the real axis of the Nyquist plot at a high frequency, utilizing the Z‐view software. Subsequently, the conductivity of the CEAB was calculated using the formula *σ* = *L* / (*A*·*R*), where *L* represents the distance between the electrodes, and *A* signifies the cross‐sectional area of the sample.

### 4.10 Evaluation of Mechanical Properties

The tensile mechanical properties were assessed through uniaxial tensile and unconfined compression tests conducted using a LISHI LE3104 setup with a 10 N loading capacity. Different thickness of 3.55, 50, 150, 250, and 500 µm CEAB film was cut to dimensions of 100 mm × 20 mm. To prevent the occurrence of cracks at the clamping positions, both ends of the sample were securely attached to two pieces of paper before being connected to the fixture. The test was conducted with a consistent peeling speed of 30 mm/min.

### 4.11 The Assessment of Interfacial Adhesion Properties

We employed a 90° peel‐off test, as introduced in Figure S15 and Movie S3 in the Supporting Information. In this evaluation, a 3.55, 50, 250, and 500 µm thick CEAB film, cut to dimensions of 100 mm × 20 mm, was securely attached to a fresh porcine skin specimen, respectively. The test was conducted with a consistent peeling speed of 30 mm/min.

### 4.12 Evaluation of Self‐Healing Property

The precursor was cured for 5 min under UV light. After the photoreaction, a piece of CEAB gel was cut into two sections, which were then covered. After 8 h, the two sections are completely self‐healed under a photomicroscope.

### 4.13 In Vitro Biocompatibility Testing

The biocompatibility of CEAB gel was assessed in vitro using the assay (CCK‐8 kit, Yeasen, Guangzhou, China). A piece of CEAB gel (2.0 g) is immersed in 10 mL of deionized water for 24 h at room temperature. Then the mouse embryonic fibroblasts cell line (NIH3T3) was plated in a 96‐well plate (2000 cells/well) with six parallel Wells (*n* = 6) in which NIH3T3 cells were cultured in Dulbecco's Modified Eagle Medium (DMEM) medium (HyClone, USA) and complete growth medium with 10% fetal bovine serum, 100 U mL^−1^ penicillin and 0.1 mg mL^−1^ streptomycin (Thermo Fisher Scientific, USA) at 37°C in 5% CO_2_. The total volume of the medium used in this work was 100 µL. After 12 h of cell culture, 10 µL CEAB gel extract was added to the medium, and the cells were then incubated at 37°C for 24 h. At the time point to be measured, CCK‐8 (10 µL), 10% of the total volume of the medium, was added and incubated for 2 h in the dark. The absorbance of the 96‐well plate was measured at 450 nm using a microplate reader (Tecan MicroplateReader Spark, USA). The cell viability is calculated according to the formula. The blank control was not seeded with NIH3T3 cells in the 96‐well plate, and the negative control was only added DMEM complete medium after seeding NIH3T3 cells in the 96‐well plate.

$$\mathrm{Cell}\;\mathrm{viability}\;\left(\%\right)=\frac{OD_{S}-OD_{C}}{OD_{N}-OD_{C}}$$

where OD_S_, OD_C_, and OD_N_ were the OD values of the samples, blank control, and negative control, respectively.

### 4.14 Live‐Dead Staining Assay

The experiment consists of three groups: control, sample 1, and sample 2. The control group received 1 mL of complete culture medium, while the sample 1 and sample 2 groups received 1 mL of their respective working solutions, with deionized water for sample 1 and CEAB extract for sample 2. NIH3T3 cells in the logarithmic growth phase were counted and adjusted to a concentration of 1 × 10^5^ cells per dish. Cells were then seeded into confocal culture dishes and incubated overnight at 37°C with 5% CO₂ to allow for adhesion. After 24 h of treatment according to the specified group, the cells were prepared for staining. For the staining solution, dye diluent (Solution C) was used to dilute Reagent A (Calcein‐AM) and Reagent B (PI) 10‐fold. A solution was freshly prepared by mixing 985.5 µL PBS, 10 µL of the diluted Reagent A, and 4.5 µL of Reagent B. To perform the live/dead staining, cells were washed once with PBS to remove any excess serum. Then, 1 mL of the staining solution was added per dish, and the cells were incubated at room temperature in the dark for 15 min. The staining process was terminated by washing the cells three times with PBS, after which the results were observed and imaged in 3D at 100× magnification.

### 4.15 Cell Recovery

The frozen storage tube containing 1 mL of cell suspension was quickly shaken and thawed in a 37°C water bath, and 4 mL of medium was added to mix well. Centrifuge at 1000 rpm for 3 min, discard the supernatant, add 1–2 mL culture medium, and then blow well. All cell suspensions were then added to a culture dish with an appropriate complete culture medium.

### 4.16 Cell Passage

Discard the upper culture medium and rinse the cells with calcium‐ and magnesium‐free PBS 1–2 times. Add 1 mL of digestion solution (0.25% Trypsin‐0.53 mM EDTA) to the culture flask, and place the culture flask in a 37°C incubator for 1 min. Then 2–3 mL of complete culture medium is added to terminate digestion. After quick, gentle mixing, transfer the contents to a sterile centrifuge tube, centrifuge at 1000 rpm for 5 min, discard the supernatant, add 1–2 mL of culture medium, and resuspend the cells with the appropriate complete culture medium in new culture dishes.

### 4.17 Water Vapor Transmission Rate (WVTR) Test

Water vapor permeability was evaluated by measuring the weight of water in a bottle where the opening was covered by the target films. Pure water (1 g) was placed in a sample bottle with a diameter of 15 mm. The CEAB gel film (3.55 µm) was attached to the opening of the bottle with an adhesive (Araldite, Nichiban). This bottle was stored in a thermostatic chamber at 25°C and a humidity of 42% for 5 days, and the subsequent decrease in weight was measured. As a comparison sample, a 4‐µm‐thick Parylene film and a 50‐µm‐thick PDMS layer film were used. A bottle without any covering was used as the reference sample.

### 4.18 Electrode/Skin Contact Impedance and Biopotential Signals Extraction

The electrode/skin contact impedance was measured by positioning two electrodes, namely commercial Ag/AgCl gel electrodes (1 mm, 1.77 cm^2^) or CEAB electrodes (3.55 µm, 1.77 cm^2^), at a distance of 7 cm on the anterior surface of a volunteer's forearm. These electrodes were then linked to the electrochemical workstation (CHI 760E) by covering the 3.55 µm CEAB film on the flat surface of a metal disk, which was then connected to a metal wire by fixing the crocodile to the metal buckle. Impedance spectra were captured within the frequency range of 1 to 10^4^ Hz, with the AC voltage amplitude fixed at 0.1 V.

The EMG signal recording setup comprises two main components: a microcontroller (Arduino UNO microcontroller) and a detector (Muscle SpikerBox Pro). The EMG signals are captured by the Spikershield box through potential differences between the working electrodes on the target area and the reference electrodes. Signal processing algorithms are applied to the collected data using Python 3.8 for fundamental signal analysis (root‐mean‐square).

The ECG data acquisition device employs a portable, wearable chest adhesive ECG sensor based on the BMD101 chip. The BMD101 chip comprises two main components: a low‐noise amplifier and an ADC (analog‐to‐digital converter). These components work in tandem, with the BMD101 chip capturing and amplifying faint bioelectrical signals originating from the heart via chest electrodes. Once the ECG signals are converted into digital form by the ADC, the BMD101 chip processes them digitally, including tasks like filtering, sampling, and data compression. Finally, the chip transmits the data to a smartphone via Bluetooth, enabling real‐time monitoring of cardiac activity and subsequent recording and analysis. The ECG signals were analyzed using the Python 3.8 envelope function.

In EMG testing, in cases recording the grip force muscle potential and collecting EMG signals during various hand gestures, two CEAB electrodes were placed on the forearm, with a CEAB film used as the reference electrode on the posterior elbow. These electrodes recorded signals generated by the brachioradialis muscle. For cases involving the collection of EMG signals during knee reflex tests, two CEAB electrodes were positioned on the anterior thigh, and a CEAB film was applied to the knee as the reference electrode. These electrodes captured signals generated by the quadriceps muscle. In cases where EMG signals during finger flexion and extension were collected, two CEAB electrodes were positioned on the forearm. In cases where facial EMG was collected when a volunteer smiled, two CEAB electrodes were attached to the risorius and zygomaticus, and a reference CEAB electrode was attached behind the ear. The Ag/AgCl gel electrodes were also attached to the same locations in the comparison cases.

The ECG signals were acquired by placing two CEAB film electrodes in specific positions: one below the clavicle, in the third intercostal space, near the heart, and the other in a position symmetrically aligned with the midline of the chest. Subsequently, these electrodes were connected to an ECG sensor based on the BMD101 chip's signal recording device. The acquired ECG signals could then be monitored in real time on a laptop and subsequently analyzed using Python's envelope function.

In EEG measurements, CEAB electrodes were positioned according to the 10–20 system for electrode placement on the head. The CEAB electrode was located at the FP1 site (at the left side of the forehead) as the working electrode, with the FPz site (at the frontal region of the brain) serving as the reference electrode. Another CEAB film electrode was placed behind the ear to serve as the ground electrode.

### 4.19 Motion Artifact Characterization

The electromechanical vibrator was placed near the working electrodes (about 2 cm) to induce skin vibration, which is similar to vibration near the chest during body movement.

### 4.20 Long‐Time Monitoring of EEG

Brain activity was assessed using EEG signals recorded with an ECG sensor based on the BMD101 chip. The working electrode was positioned at the FP1 site, the reference electrode at the FPz site, and the ground electrode behind the ear. During the 12‐hour monitoring period, the volunteer slept for 1 h, engaged in 0.5 h of exercise, and finally returned to a state of calm without removing the CEAB electrodes. Subsequently, the EEG signal is analyzed using Python 3.8 and fast Fourier transformation (FTT).

### 4.21 Depression Detection

Depression data collection: We recruited six depressed and six healthy volunteers from the Fifth Affiliated Hospital of Wenzhou Medical University in China (all the depressed volunteers were diagnosed by the Hamilton Depression Scale in the hospital). EEG signals were recorded by a portable TGAM EEG device with a single channel. The ring‐shaped EEG sensor was tied to the head, and all people were asked to keep calm and keep their eyes closed for 1 min without any interruption.

Depression data preprocess: Building upon the TGAM module, the embedded algorithms filtered power line frequency noise. The dataset encompassed both the raw EEG original values and transformed values across various frequency bands. The data were first cleaned by deleting invalid “0” values received at the beginning stage, then aligned following the sampling frequency of 512 Hz. A 2‐s duration of data was set as a sample, with an overlap of 50%.

Features extraction: Twelve linear features including original value mean, original value *σ* (standard deviation), attention mean, attention *σ*, *δ* mean, *θ* mean, low *α* mean, high *α* mean, low *β* mean, high *β* mean, low *γ* mean, mid *γ* mean, low *β* mean / *θ* mean, high *β* mean / *θ* mean, and two nonlinear features including C0 complexity and power spectral entropy were extracted as features for normal, depression classification.

Machine learning models construction: SVM, KNN, XGBOOST, and RF were constructed by the sklearn toolkit, Python 3.8.

### 4.22 Hand Gesture Replication by Robotic Hands

Gesture dataset collection: Ten volunteers are instructed to repeat six different gestures—six, eight, good, yeah, ok, and fist—each lasting 5 s, with a 5‐s rest between consecutive gestures to prevent muscle fatigue. This entire sequence is collected over 1 min and repeated for ten volunteers.

Data preprocess: The gesture data is first sliced at the length of 1000, overlapped at 95%, normalized, and then reshaped as 20 × 50 dimension.

CNN model construction: The CNN architecture is established by the Keras toolkit in Python 3.8.

## Author Contributions

Conceptualization: Zhenglin Chen, Likun Zhang, Dongmei Yu, and Peiwu Qin. Methodology: Likun Zhang, Huazhang Ying, Zhicheng Du, Ziwu Song, and Jiaju Chen. Investigation: Zhenglin Chen and Likun Zhang. Visualization: Likun Zhang, Zhenglin Chen, Huazhang Ying, Xi Yuan, Ping Zhang, Jiaju Chen, and Peisheng He. Supervision: Dongmei Yu, Peiwu Qin, Zhenglin Chen, and Chenggang Yan. Writing—original draft: Likun Zhang and Zhenglin Chen. Writing—review and editing: Zhenglin Chen, Peiwu Qin, Wenbo Ding, Canhui Yang, Vijay Pandey, Xinhui Xing, Jiansong Ji, Chenggang Yan, and Liwei Lin.

## Ethics Statement

The study protocol was thoroughly reviewed and approved by the ethical committee of the Tsinghua Shenzhen International Graduate School, Tsinghua University (approval number 2023‐F036).

## Conflicts of Interest

The authors declare no conflicts of interest.

## Supporting information



Supporting information

Supporting information

Supporting information

Supporting information

Supporting information

Supporting information

## Data Availability

The data that support the findings of this study are available from the corresponding author upon reasonable request.
