# Supplementary material for: A 3.55‐µm Ultrathin, Skin‐Like Mechanoresponsive, Compliant, and Seamless Ionic Conductive Electrode for Epidermal Electrophysiological Signal Acquisition and Human‐Machine Interaction
Source: Exploration (Beijing). 2025 Jul 8;5(5):20240232. doi: 10.1002/EXP.20240232 (PMC12561302; doi:10.1002/EXP.20240232)
Supplement: Supplementary file 1 — Supporting information [file EXP2-5-20240232-s003.docx]

Supporting Information

A 3.55-μm ultrathin, skin-like mechanoresponsive, compliant, and seamless ionic conductive electrode for epidermal electrophysiological signal acquisition and human-machine-interaction

Likun Zhang^1,3,4,9,10#^, Peiwu Qin^2,3,8,9^*, Huazhang Ying^1,4^, Zhicheng Du^3,4,5^, Chenying Lu^2^, Minjiang Chen^2^, Liyan Lei^2^, Ziwu Song^1,3^, Jiaju Chen^1,4^, Xi Yuan^1,3,4^, Canhui Yang^6^, Vijay Pandey^1,3,4^, Can Yang Zhang^4,5^, Dongmei Yu^7^, Peisheng He^10^, Liwei Lin^3,10^, Wenbo Ding^1,3^, Xinhui Xing^1,4,5^, Chenggang Yan^8,9^*, Jiansong Ji^2*^, Zhenglin Chen^2,3,8,9#^*

1. Shenzhen International Graduate School, Tsinghua University, Shenzhen, China
2. Zhejiang Key Laboratory of Imaging and Interventional Medicine, Zhejiang Engineering Research Center of Interventional Medicine Engineering and Biotechnology, The Fifth Affiliated Hospital of Wenzhou Medical University, Lishui 323000, China
3. Tsinghua-Berkeley Shenzhen Institute, Tsinghua Shenzhen International Graduate School, Tsinghua University, Shenzhen, China
4. Institute of Biopharmaceutical and Health Engineering, Shenzhen International Graduate School, Tsinghua University, Shenzhen, Guangdong, China
5. Key Lab for Industrial Biocatalysis, Ministry of Education, Department of Chemical Engineering, Tsinghua University, Beijing 100084, China
6. Soft Mechanics Lab, Department of Mechanics and Aerospace Engineering, Southern University of Science and Technology, Shenzhen, China
7. School of Mechanical, Electrical & Information Engineering, Shandong University, Weihai, Shandong, China.
8. School of Automation, Hangzhou Dianzi University, Hangzhou, Zhejiang Province, 310018, China
9. Lishui Institute of Hangzhou Dianzi University, Lishui, Zhejiang Province, 323000, China
10. Department of Mechanical Engineering, University of California, Berkeley, CA 94720, United States of America

# These authors contributed equally to this work and should be considered as co-first authors.

* Corresponding author's emails: [zhenglin.chen@sz.tsinghua.edu.cn](mailto:zhenglin.chen@sz.tsinghua.edu.cn); [jjstcty@sina.com](mailto:jjstcty@sina.com); [pwqin@sz.tsinghua.edu.cn](mailto:pwqin@sz.tsinghua.edu.cn).

Supplementary Movies

**Movie S1.** Precursor diffusion (Online).

**Movie S2.** Self-healing process of CEAB (Online).

**Movie S3.** CEAB is peeled off from the porcine skin (Online).

**Movie S4.** Finger movements recorded by CEAB electrodes (Online).

**Movie S5.** Six, eight, good gesture repetition by CEAB electrodes (Online).

Supporting Figures


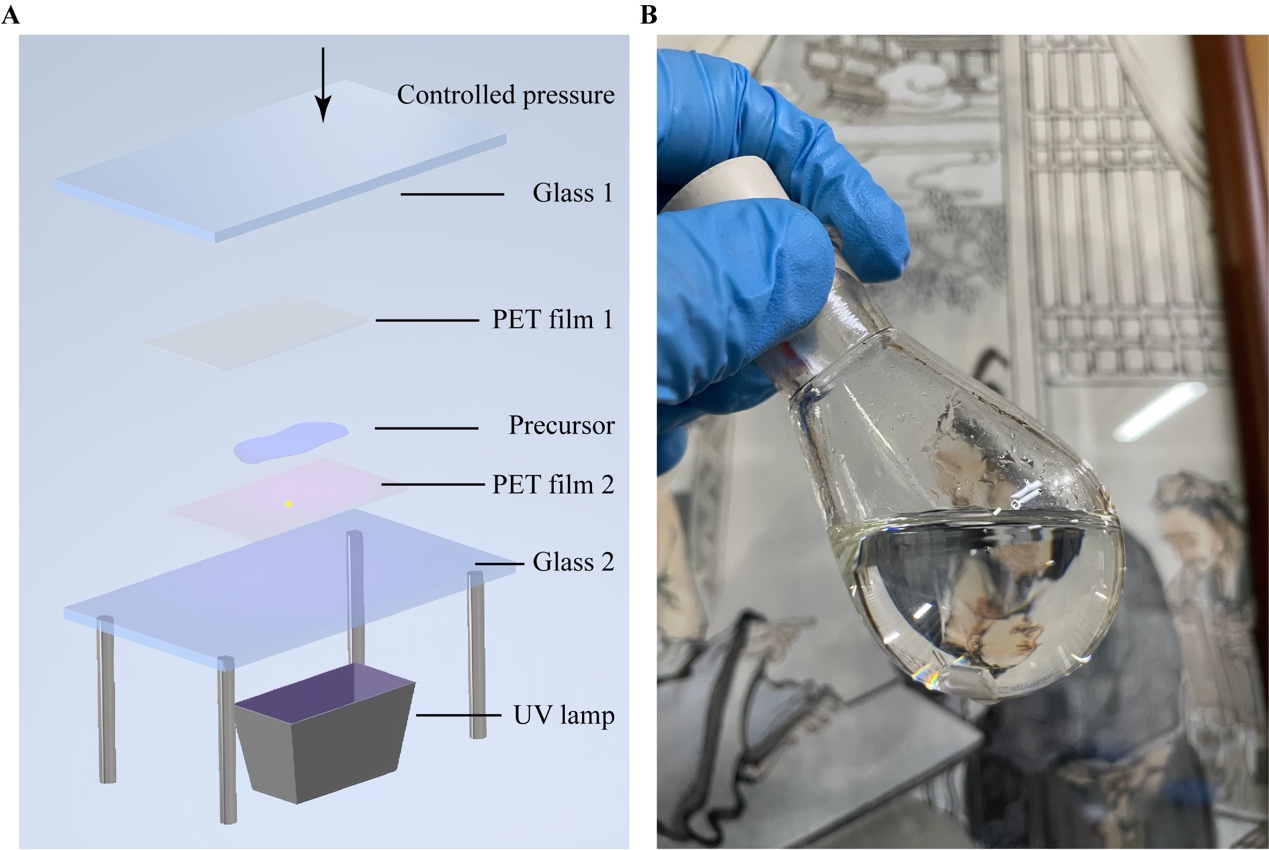


Figure S1. Structure of the device and precursor solution. A**,** The setup of the device for preparing ultrathin CEAB film. B**,** The transparent precursor solution.


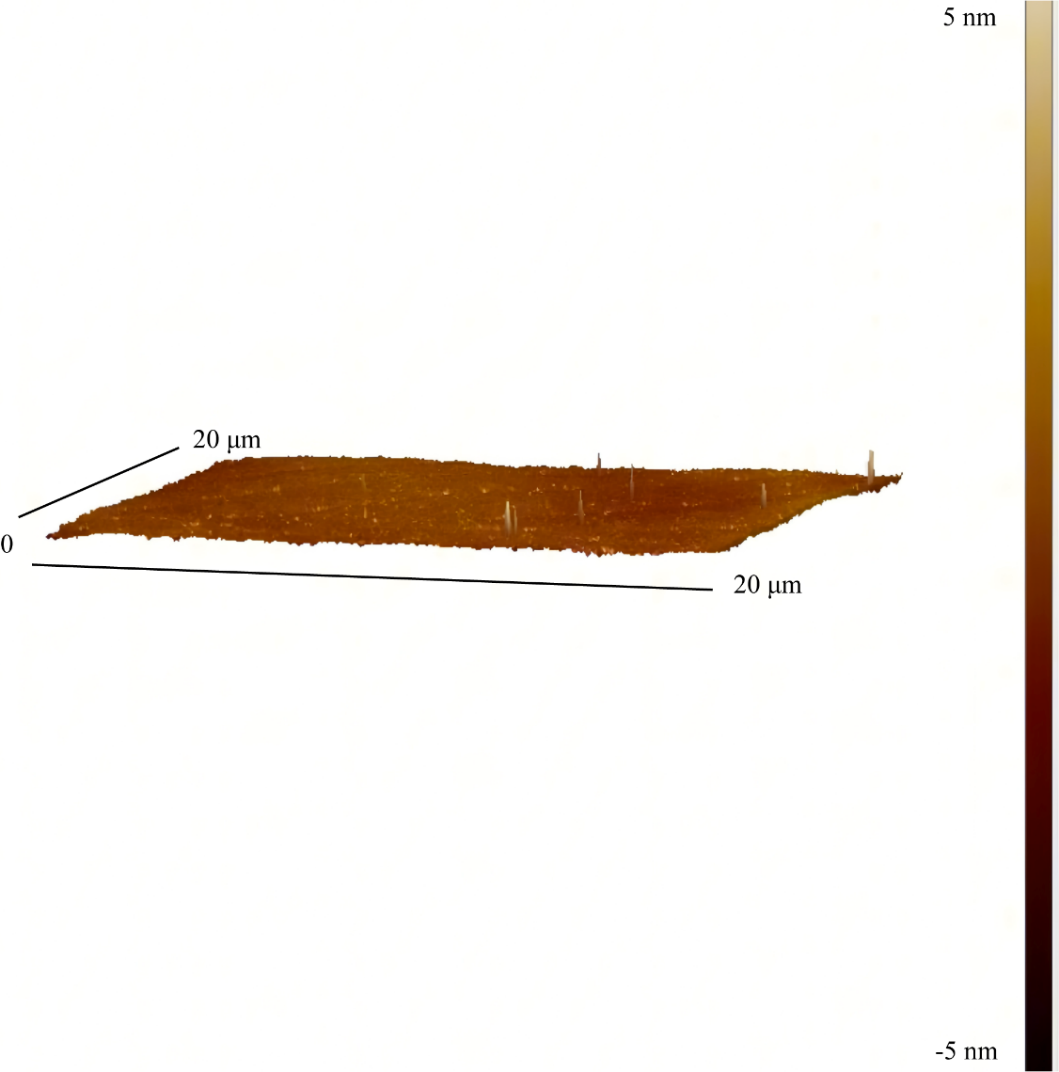


**Figure S2.** AFM height 3D image of CEAB film.


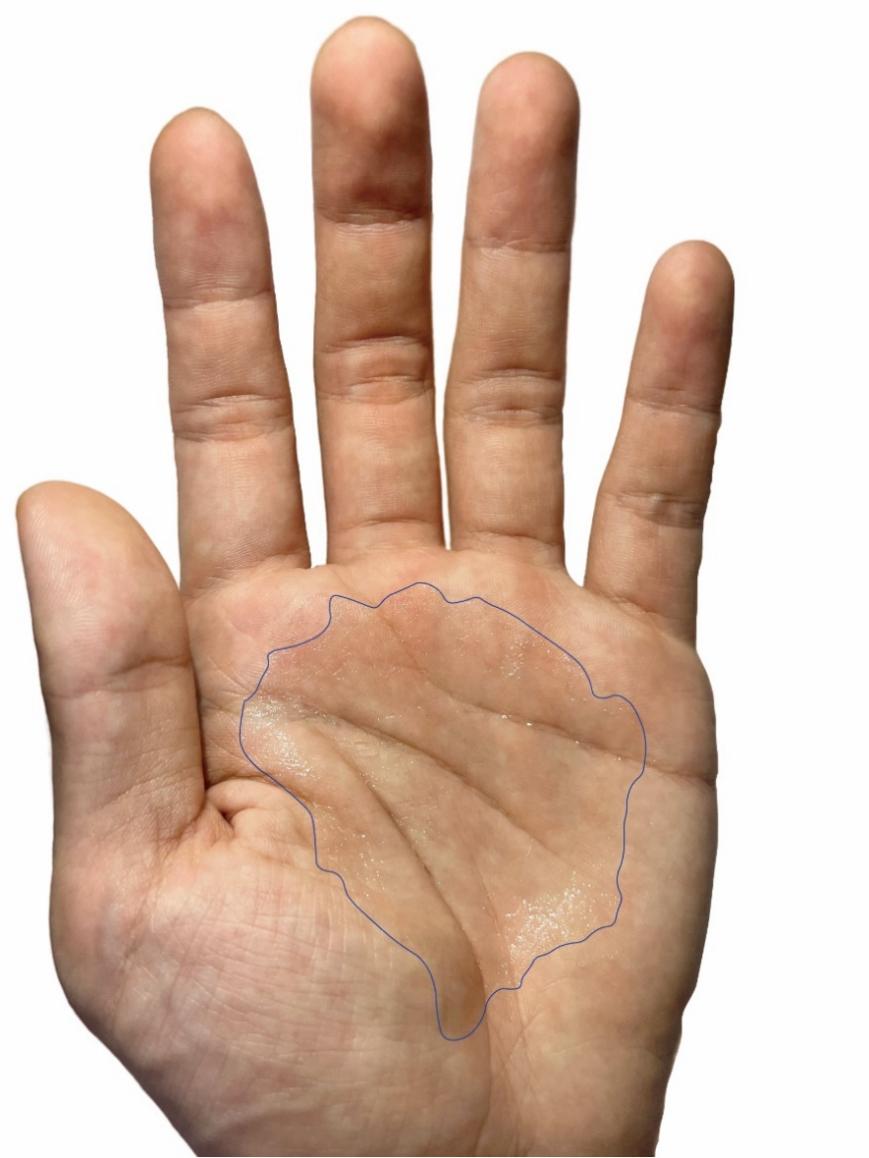


**Figure S3.** The 3.55 μm thick CEAB film is applied to the palm, which can effectively replicate the complex and curved rough surface across a large area.


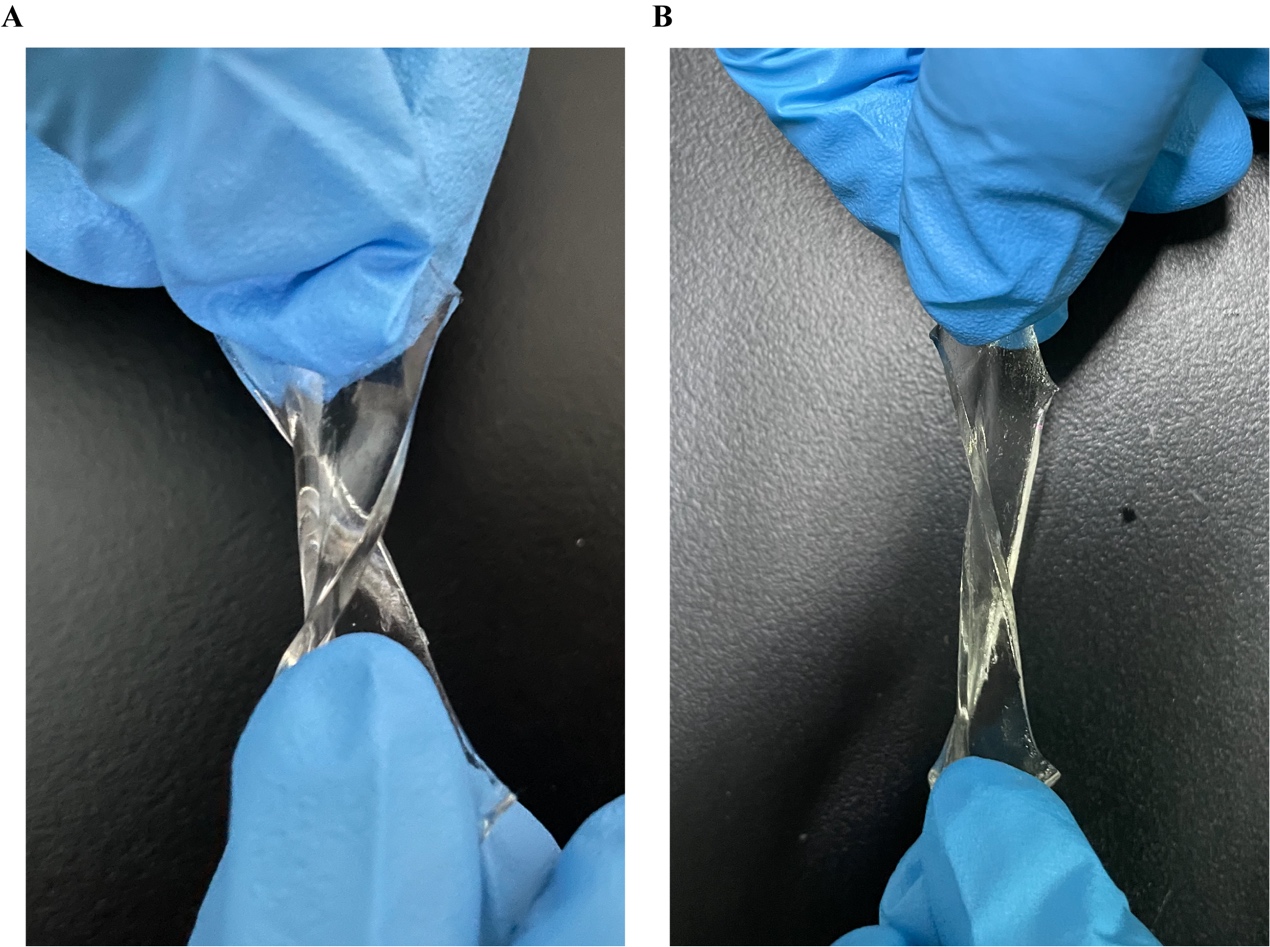


**Figure S4.** CEAB film is flexible and twisted at A, 25 ℃, and B, after stored at -30 ℃ for 1 h.


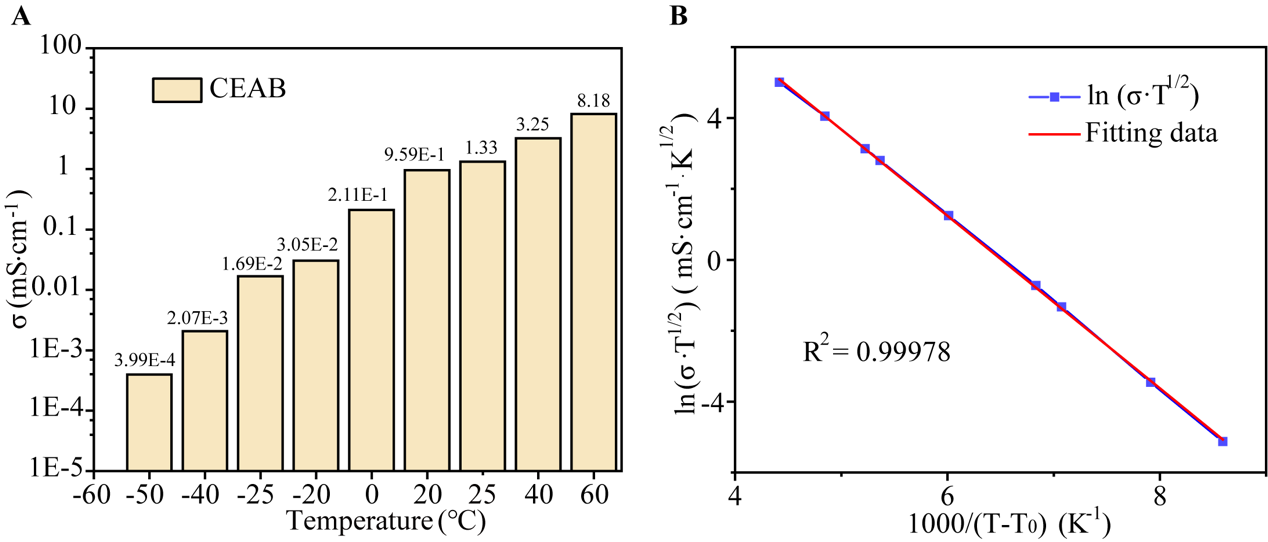


**Figure S5.** A, CEAB film conductivity at different temperatures. B, The relationship between ionic conductivity and temperature follows the Vogel-Fulcher-Tammann (VFT) relationship. The fitted data is consistent with the experimental data.


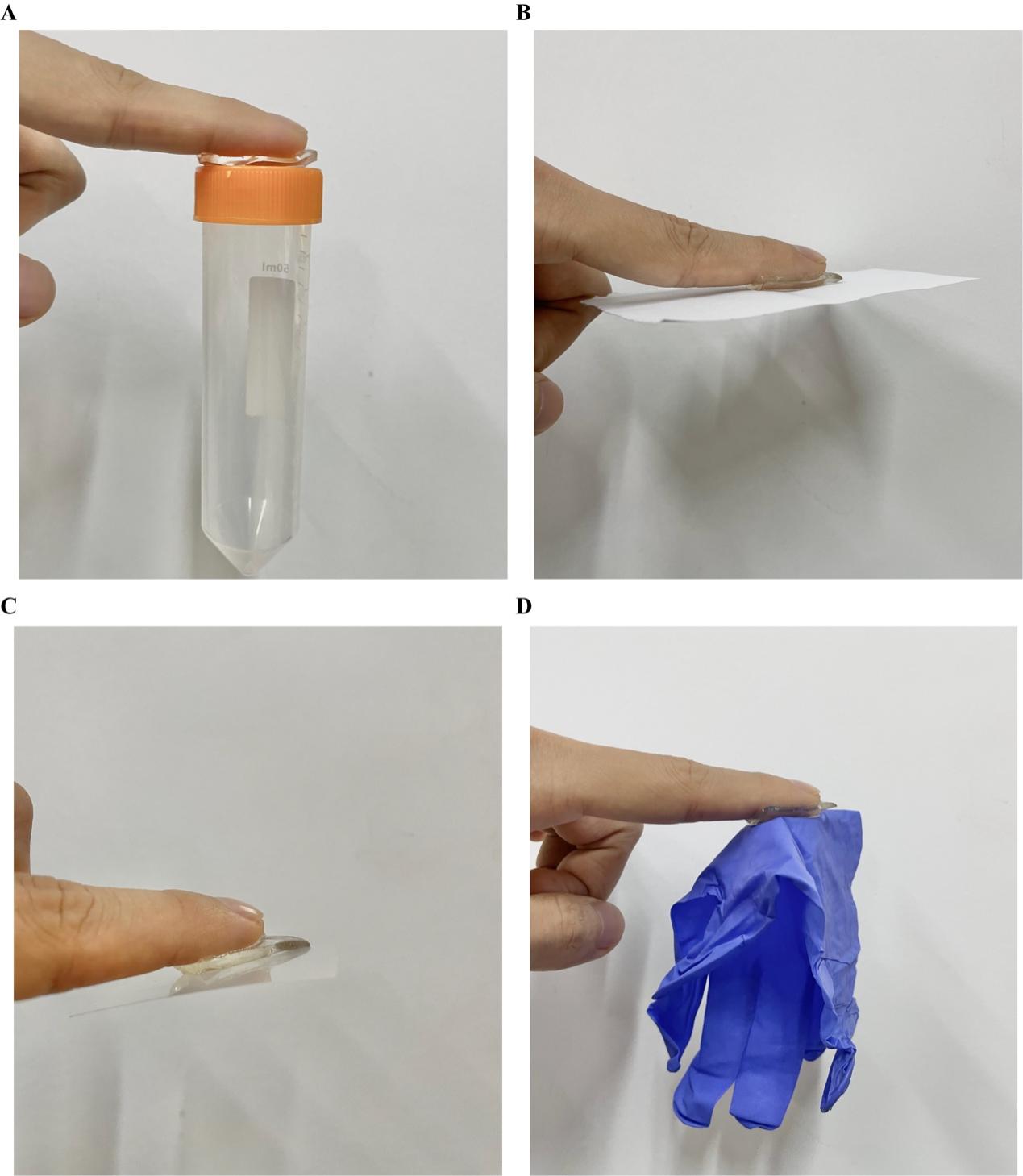


**Figure S6.** Adhesive behaviors of 2 mm CEAB film to various substrates A, plastic tube B, paper (cellulose) C, PET film D, rubber glove.


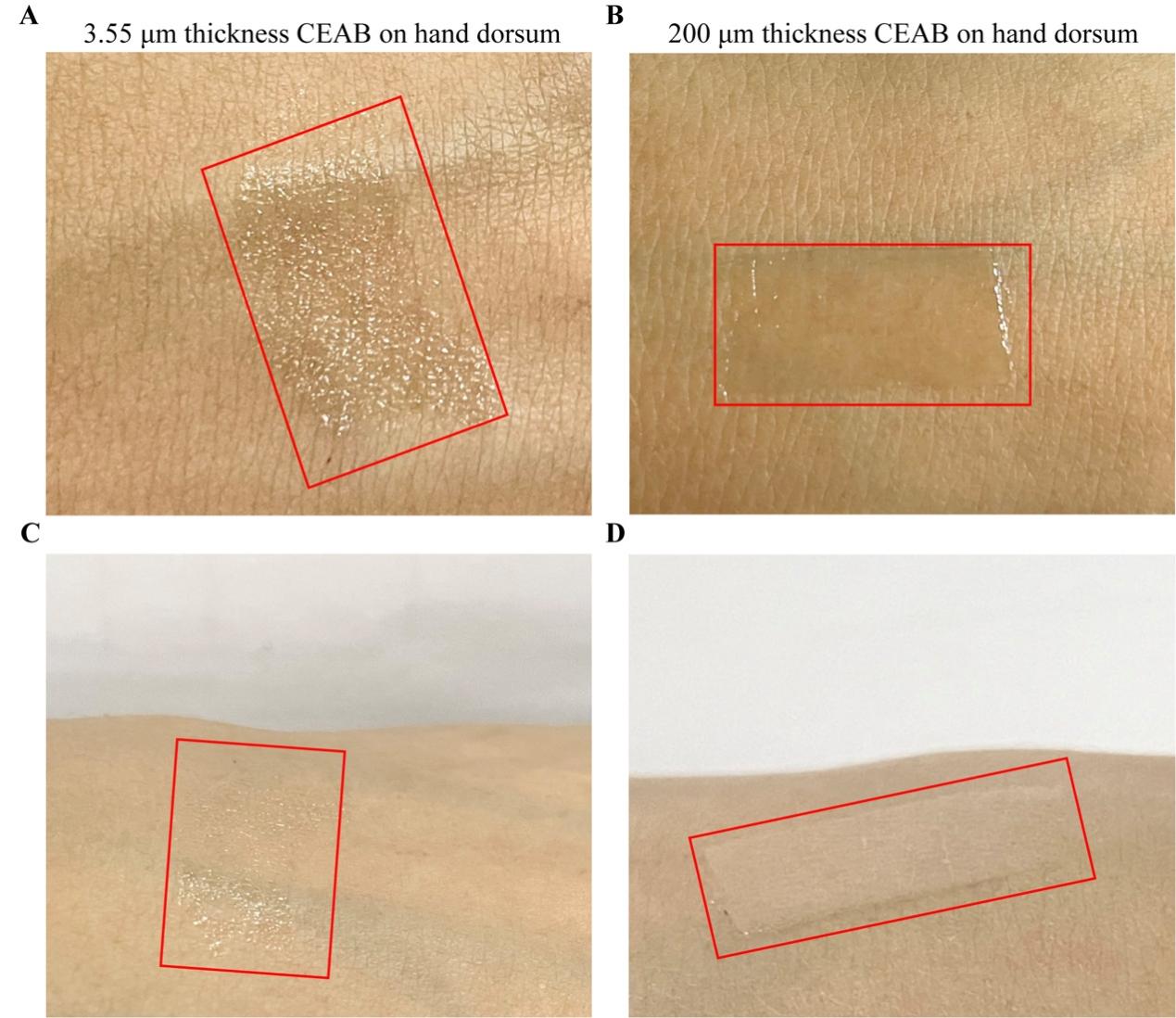


**Figure S7.** The digital photograph of 3.55 (A and C), and 200 (B and D) μm thick CEAB film adherent to human skin closely.


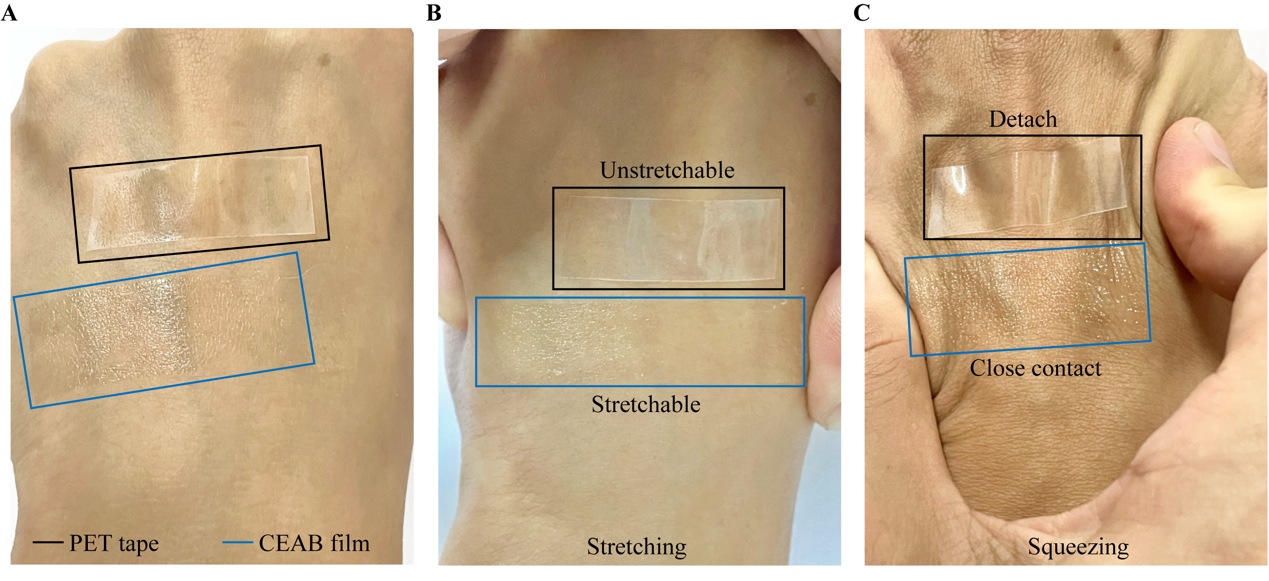


**Figure S8.** Digital images of 3.55 μm CEAB (blue) film A, original and under deformation behaviors induced by B, stretching, and C, squeezing the local skin. A 10 μm PET (black) tape is used as a contrast.


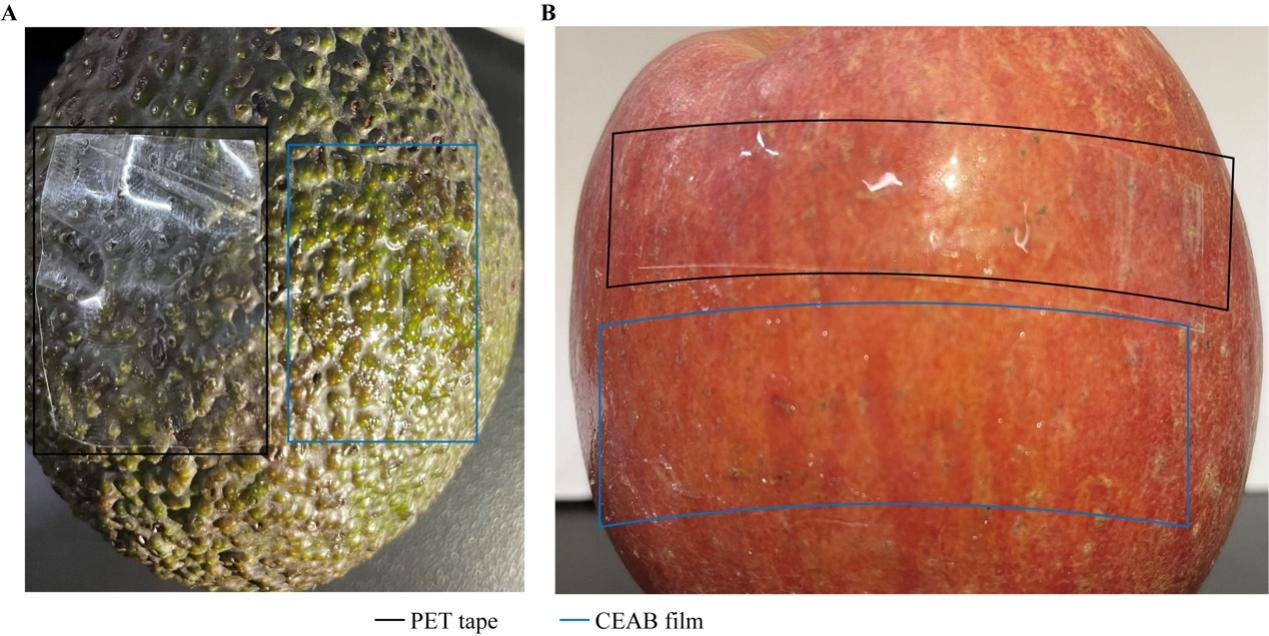


**Figure S9.** Conformal behavior of 3.55 μm CEAB (blue) film adherent to fruits with different surface roughness. A, avocado, and B, apple. A PET (black) tape is used as a contrast.


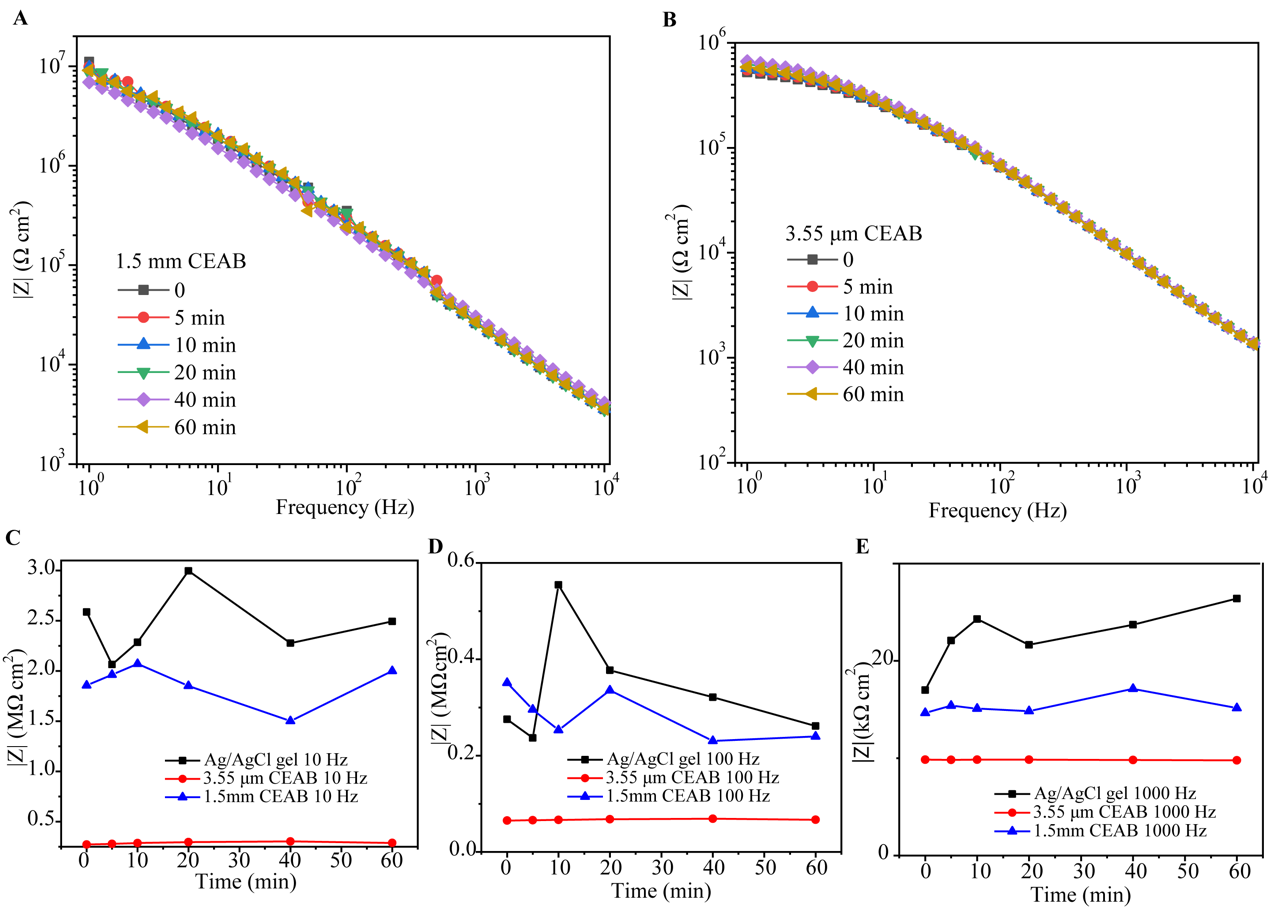


**Figure S10.** CEAB electrode skin contact impedance. Impedance changes of CEAB electrode attached to the skin for 1 h of A ,1.5 mm and B, 3.55 μm, respectively. C-E, CEAB, and Ag/AgCl gel electrode/skin contact impedance at 10, 100, and 1000 Hz respectively.


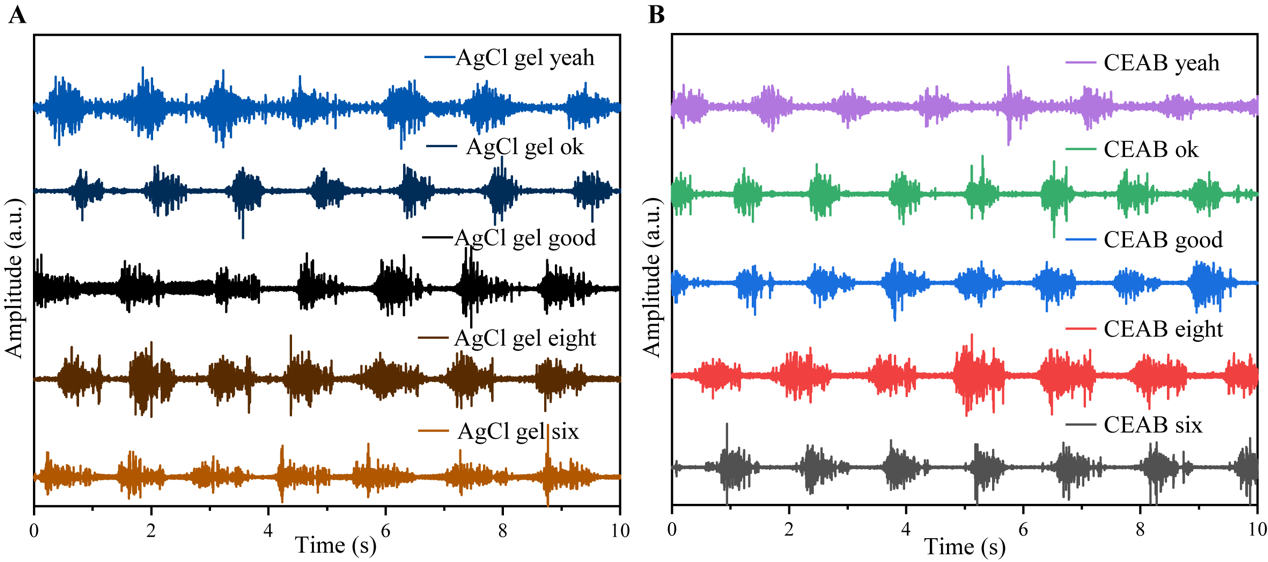


**Figure S11** The EMG biopotential of six gestures collected by A, AgCl/Ag gel electrode and B, CEAB electrodes.


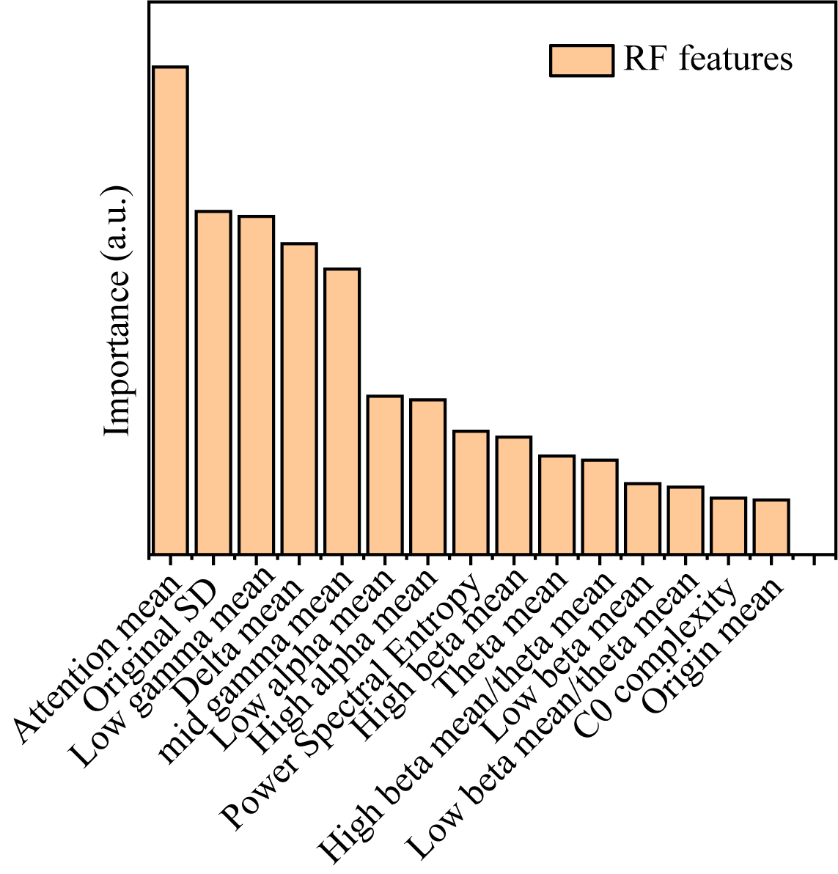


**Figure S12** Individual feature importance contributing to RF models for depression classification.


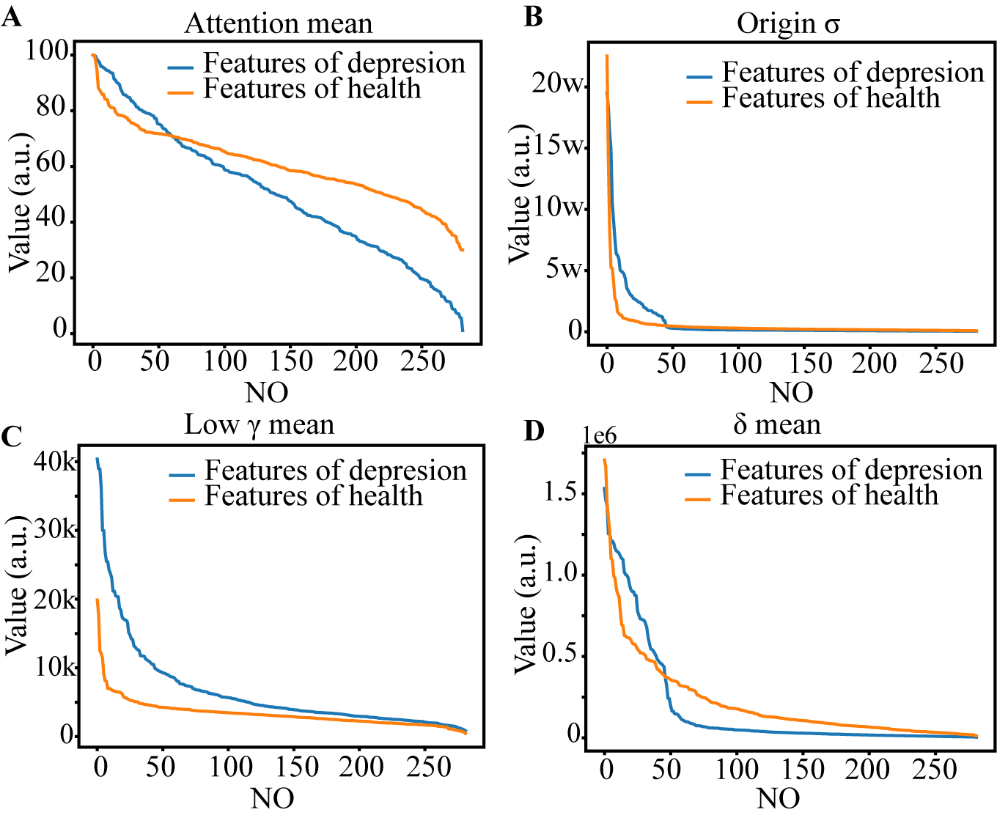


**Figure S13** Statistical histogram by visualizing the relationship of feature values and numbers in terms of the 4 paramount feature types: A, attention mean, B, origin σ C, low γ mean D, δ mean.


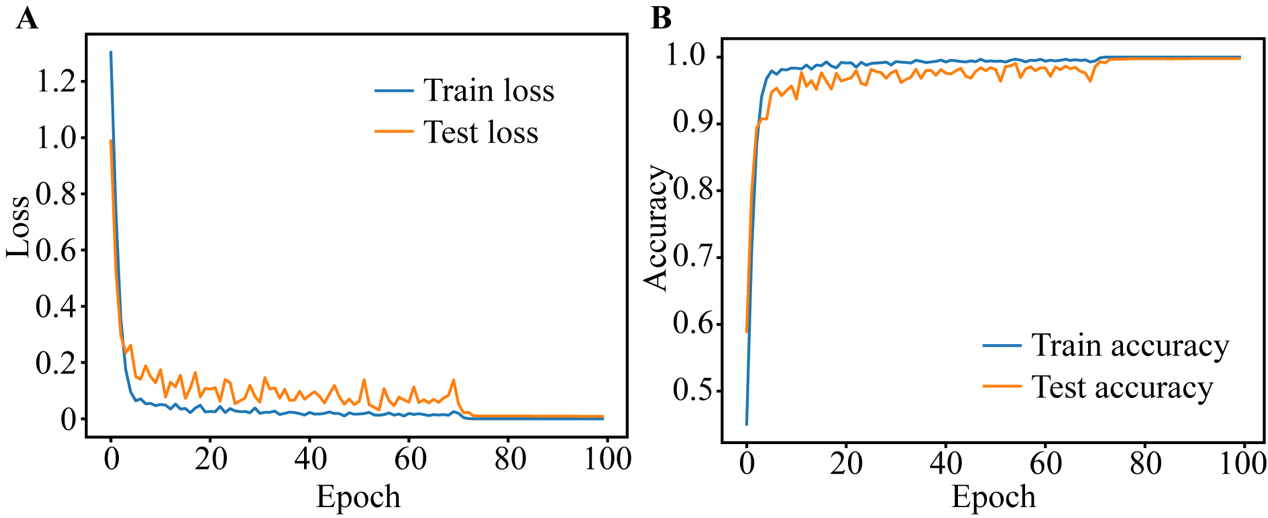


**Figure S14** Training process of the CNN algorithm. A, Loss, and B, the accuracy of the training set and test set during the training process of the CNN algorithm for gesture recognition.


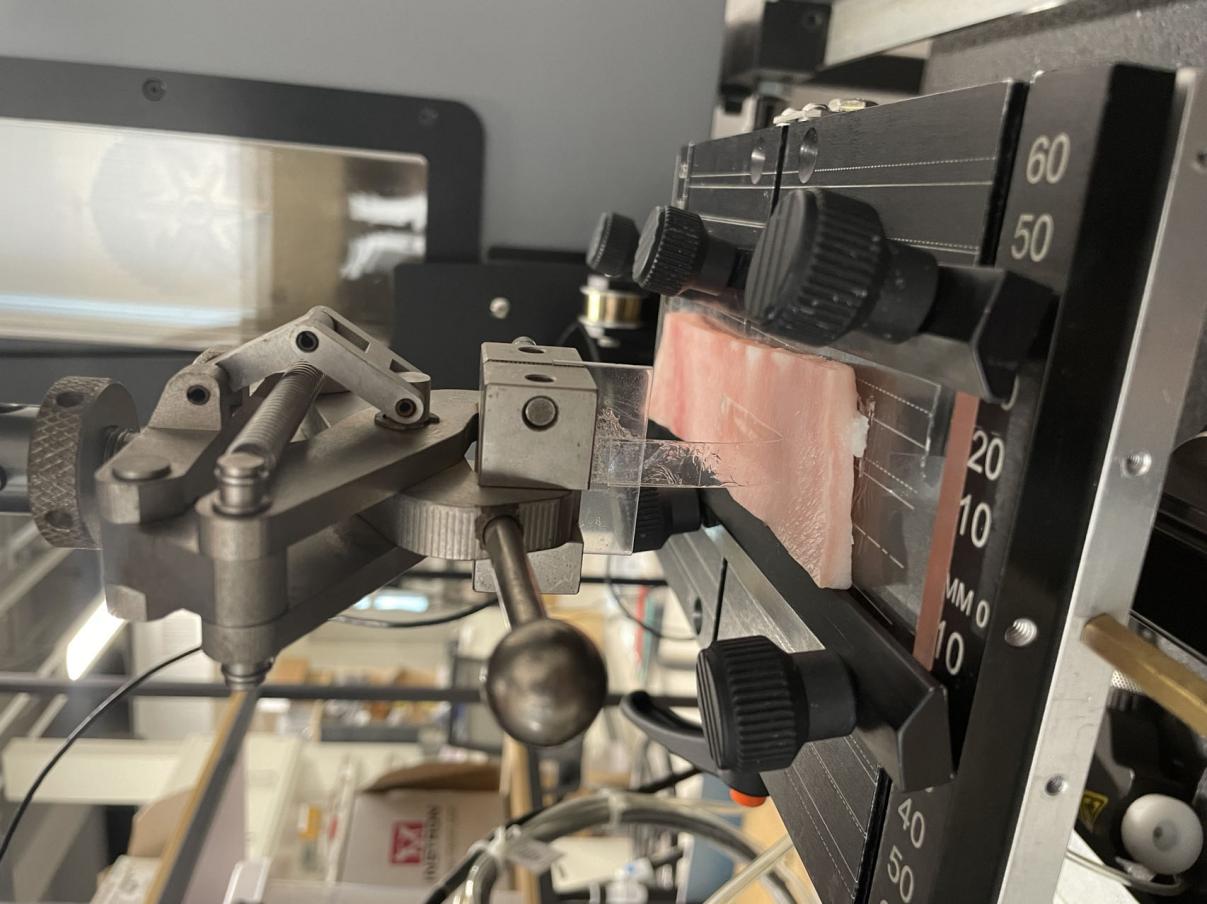


**Figure S15** A photograph illustrates the configuration employed for the 90-degree peeling test. A 100 μm thick PET is glued on the other surface of the CEAB film to prevent elongation in the peeling direction.


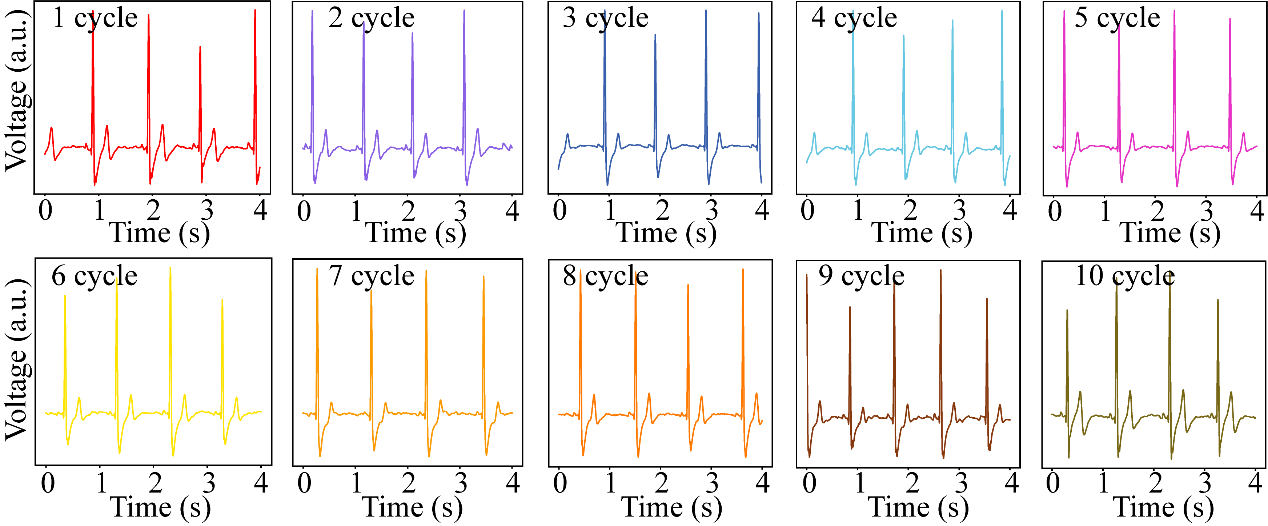


**Figure S16** CEAB electrodes for reusable ECG signal monitoring. 3.55 μm CEAB electrodes monitor ECG signals during 10 consecutive touch/detouch cycles.


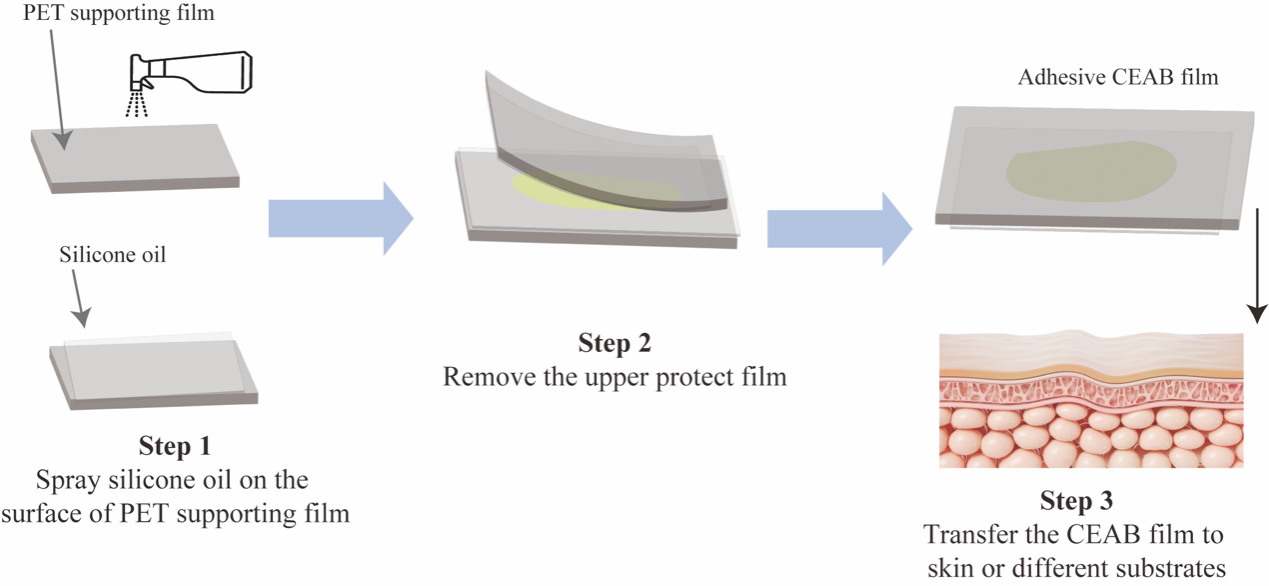


Figure S17 Transfer process of CEAB film to the substrate.


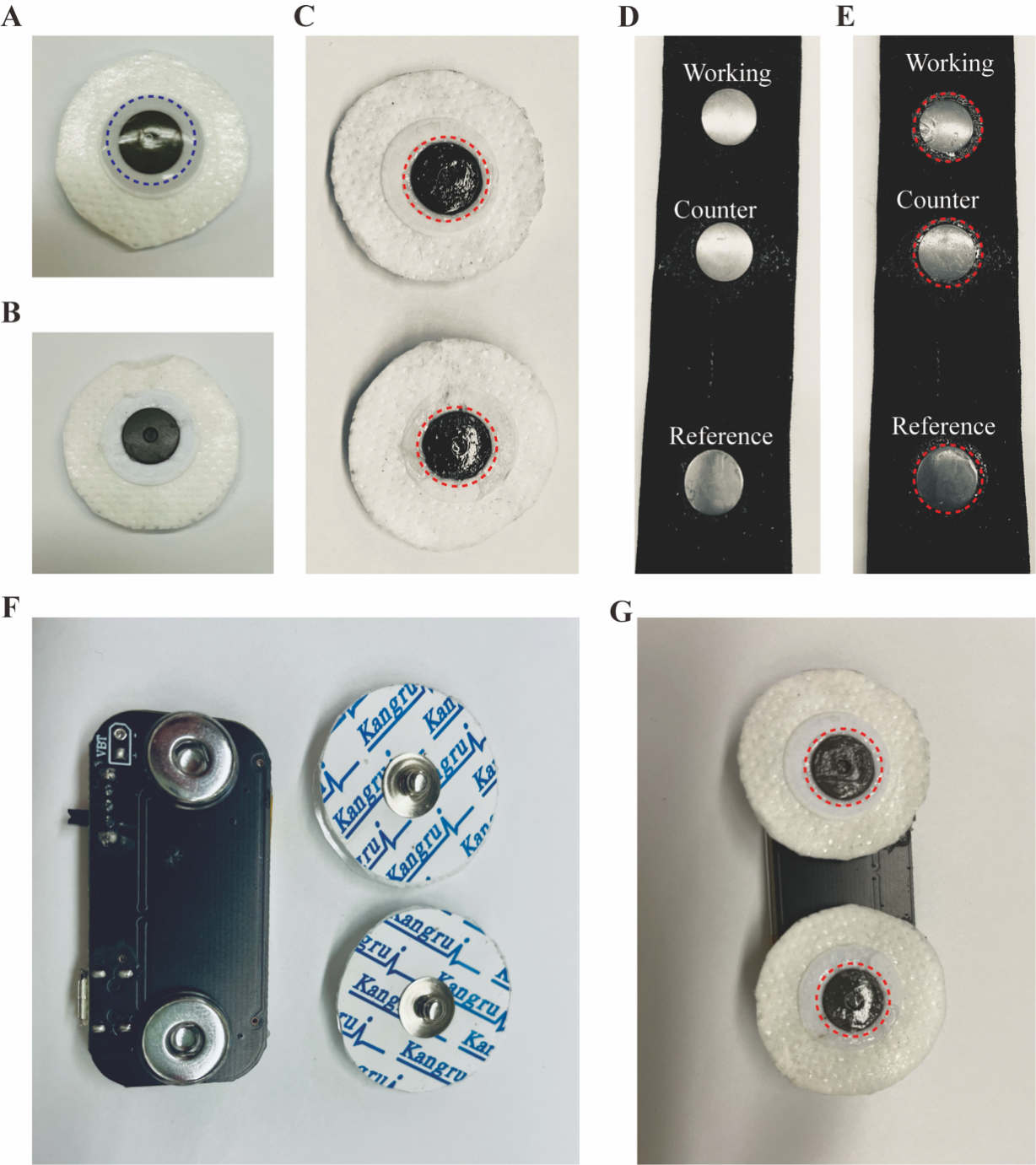


Figure S18 CEAB film-based electrodes and corresponding electrical connection. A, commercial Ag/AgCl electrode with a hydrogel adhesive layer. B, Ag/AgCl electrode without hydrogel layer. C, CEAB film-based electrode for recording EMG/ECG signals. D, the commercial metal electrode of a wearable EEG headband. E, CEAB films covered the electrode of an EEG headband. ECG acquisition sensor before (F) and after (G) assemble.


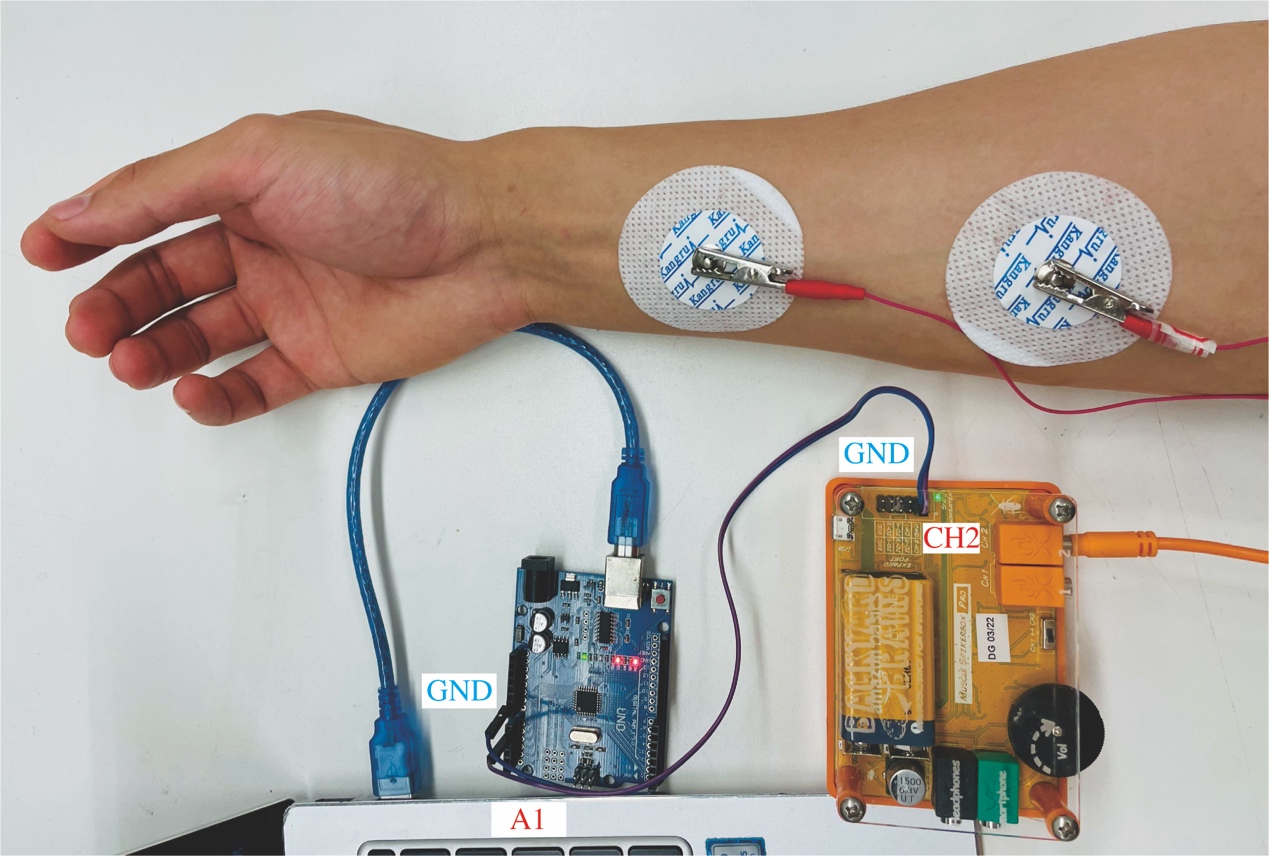


Figure S19 Electrical connection of Spikerbox Pro to Arduino Uno for ADC data acquisition.


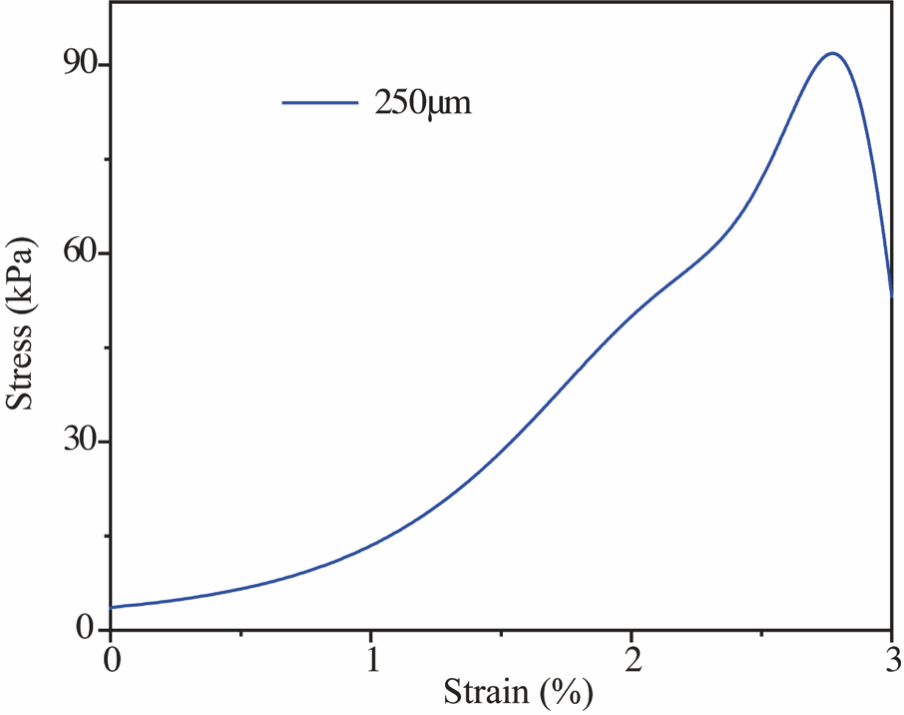


Figure S20 Mechanical stretch strain-stress curve of 250 μm CEAB film at 40% RH.

**Supporting Table 1** Comparison of the practical applications of various types of electrodes for electrophysiology.

| Sample | ECG | EMG | EEG | Wearable disease detection | Human-machine-interaction | Ref |
| --- | --- | --- | --- | --- | --- | --- |
| AgPH | Higher SNR ECG recording both in static and exercise state | No | No | No | No | 10.1007/s42114-022-00596-y |
| IG-50 | Higher SNR ECG recording, with comparison to Ag/AgCl in the static state | No | No | No | No | 10.1039/C5TC01888A |
| PTG | Higher SNR and lower noise voltage vs. Ag/AgCl both in static and dynamic status | Higher SNR and lower noise voltage vs. Ag/AgCl both in static and dynamic status | EEG monitoring for ~12 hours | No | Multichannel, four gestures repetition by robotic hands | 10.1038/s41467-021-25152-y |
| PWS | Lower noise voltage vs. Ag/AgCl both in static and dynamic status | No comparison with other electrodes | EEG record during sound stimuli | Atrial fibrillation detection with the absence of P wave | Open hands and close hands repetition of artificial limbs | 10.1038/s41467-020-18503-8 |
| CEAB | Higher SNR vs Ag/AgCl gel both in the static and dynamic state | Higher SNR and lower RMS noise vs Ag/AgCl for ~3.5 h | ~12 h EEG monitoring during exercise, rest, sleep | Spinal pathway health detection with thigh EMG signals during knee jerk reflex. Depression detection with explainable EEG digital biomarkers | Single channel, finger movement record, six types of hand gesture recognition, and repetition of artificial limbs | This work |

**Supporting Table 2** Young’s modulus of CEAB film with different thickness

| Thickness (μm) | Young’s modulus (kPa) |
| --- | --- |
| 3.55 | 59.74 $\pm$ 0.23 |
| 50 | 3.99 $\pm$ 0.01 |
| 150 | 3.97 $\pm$ 0.01 |
| 250 | 3.90 $\pm$ 0.01 |
| 500 | 3.23 $\pm$ 0.01 |

**Supporting Table 3** Comparison of our electrode with representative ionic gels

| Sample | Self-healing | Skin-like | Thickness (μm) | Visible light transmittance (%) | σ (mS/cm) | Adhesion (J/m2) | λ (%) | Ref |
| --- | --- | --- | --- | --- | --- | --- | --- | --- |
| gelatin-supported DES gel | No | No | 500 | 80-90 | 2.5 | - | ~300 | https://doi.org/10.1039/C8TC05918G |
| PEA and the solvent [BMMIm][TFSI] | No | No | 1000 | >90 | 0.1 | - | >800 | 10.34133/2020/2505619 |
| PAAc–DES gel | No | No | 1000 | ~92 | 1.26 | 100 (too much) | >1000 | 10.1038/s41528-021-00118-8 |
| SAP | No | No | 500 | 93 | > 1 | < 0.0127 (too less) | <304 | 10.1021/acsami.8b18947 |
| CEAB | Yes | Yes | 3.55 | ~100 | 1.33 | ~20 (mild strong) | ~800 | This work |

Supporting codes 1:

The Arduino code for transmitting the data is listed as the following:

int b = 0; // Define variable b, for storing the assembled value

void setup()

{

Serial.begin(115200); // Open serial communication, set the baud rate to 115200

}

short int len = 200; // Define the number of samples (200 samples per loop)

void loop()

{

for (int i = 0; i < len; i++) {

b = analogRead(A1); // Read the analog value from pin A1

Serial.print(b); // Print the analog value to the serial monitor

Serial.print(','); // Add a comma as a separator after each value

}

Serial.println(); // Print a newline after one set of data

delay(1); // Set the data refresh rate to 1 millisecond

}

Supporting codes 2:

The Python 3.8 code for acquiring the data is listed as the following:

import numpy as np

import serial

import time

# Initialize serial connection, specify COM port and baud rate

ser = serial.Serial('COM8', 115200)

def read_serial_data():

timestamps = []

list_data = []

start_time = time.time() # Record the start time of data collection

curr_time = 0

while True:

if ser.inWaiting(): # Check if there is data available in the serial port

data = ser.readline().strip() # Read a line of data and remove the trailing newline character

parts = data.split(b',') # Split the data into multiple parts by commas

parts = parts[0:-1] # Remove the last empty value

for part in parts:

try:

number = part.decode('utf-8') # Decode the binary data to a string

if number.isnumeric(): # Check if the data is numeric

number = int(number) # Convert to an integer

curr_time = time.time() - start_time # Calculate the current timestamp

list_data.append(number) # Store the read value

timestamps.append(curr_time) # Store the corresponding timestamp

# Save the timestamp and data to a file

with open('./my_data/{}.txt'.format(name), 'a') as file:

file.write(str(curr_time) + ',' + str(number) + '\n')

# Print the current timestamp and value

print('==========================================\n'

'Timestamp: {}\nValues: {}'.format(curr_time, number))

except ValueError:

print('Could not convert part to integer:', part)

return start_time, timestamps, list_data

if __name__ == "__main__":

name = 'gesture' # Name of the data file

start_time, timestamps, list_data = read_serial_data()

Reference

1. Wang, B. and A.J.A.M. Facchetti, Mechanically flexible conductors for stretchable and wearable e‐skin and e‐textile devices. 2019. **31**(28): p. 1901408.

2. Bihar, E., et al., Fully printed electrodes on stretchable textiles for long‐term electrophysiology. 2017. **2**(4): p. 1600251.

3. Chen, Z., Y.J.S. Wang, and Diagnostics, *Organic ionic fluid-based wearable sensors for healthcare.* 2022. **1**(4): p. 598-613.

4. Zhao, Y., et al., Ultra-conformal skin electrodes with synergistically enhanced conductivity for long-time and low-motion artifact epidermal electrophysiology. 2021. **12**(1): p. 4880.

5. Kim, T.H., et al., Flexible biomimetic block copolymer composite for temperature and long-wave infrared sensing. 2023. **9**(6): p. eade0423.

6. Cheng, L., et al., Recent advances in flexible noninvasive electrodes for surface electromyography acquisition. npj Flexible Electronics, 2023. **7**(1): p. 39.

7. Yuk, H., J. Wu, and X.J.N.R.M. Zhao, *Hydrogel interfaces for merging humans and machines.* 2022. **7**(12): p. 935-952.

8. Hao, Y., et al., A Stretchable, Breathable, And Self‐Adhesive Electronic Skin with Multimodal Sensing Capabilities for Human‐Centered Healthcare. 2023. **33**(44): p. 2303881.

9. Lim, C., et al., Stretchable conductive nanocomposite based on alginate hydrogel and silver nanowires for wearable electronics. 2019. **7**(3).

10. Wang, J., et al., Ultrastretchable E‐Skin Based on Conductive Hydrogel Microfibers for Wearable Sensors. 2023: p. 2305951.

11. Xun, X., et al., Highly robust and self-powered electronic skin based on tough conductive self-healing elastomer. 2020. **14**(7): p. 9066-9072.

12. Fernandez, M., R.J.B.i. Pallas-Areny, and technology, *Ag-AgCl electrode noise in high-resolution ECG measurements.* 2000. **34**(2): p. 125-130.

13. De Luca, C., et al., *Pasteless electrode for clinical use.* 1979. **17**: p. 387-390.

14. Shen, G., et al., A fully flexible hydrogel electrode for daily EEG monitoring. 2022. **22**(13): p. 12522-12529.

15. Zou, Y., et al., Automatic identification of artifact-related independent components for artifact removal in EEG recordings. 2014. **20**(1): p. 73-81.

16. Zainal, S.H., et al., Preparation of cellulose-based hydrogel: A review. 2021. **10**: p. 935-952.

17. Ohm, Y., et al., An electrically conductive silver–polyacrylamide–alginate hydrogel composite for soft electronics. 2021. **4**(3): p. 185-192.

18. Hu, C., et al., Stable, strain-sensitive conductive hydrogel with antifreezing capability, remoldability, and reusability. 2018. **10**(50): p. 44000-44010.

19. Zhao, X., et al., Bioinspired ultra-stretchable and anti-freezing conductive hydrogel fibers with ordered and reversible polymer chain alignment. 2018. **9**(1): p. 3579.

20. Smith, E.L., A.P. Abbott, and K.S.J.C.r. Ryder, *Deep eutectic solvents (DESs) and their applications.* 2014. **114**(21): p. 11060-11082.

21. Stefanovic, R., et al., Nanostructure, hydrogen bonding and rheology in choline chloride deep eutectic solvents as a function of the hydrogen bond donor. 2017. **19**(4): p. 3297-3306.

22. Zhao, B.-Y., et al., Biocompatible deep eutectic solvents based on choline chloride: characterization and application to the extraction of rutin from Sophora japonica. 2015. **3**(11): p. 2746-2755.

23. Paiva, A., et al., Natural deep eutectic solvents–solvents for the 21st century. 2014. **2**(5): p. 1063-1071.

24. Lai, C.-W., S.-S.J.A.a.m. Yu, and interfaces, 3D printable strain sensors from deep eutectic solvents and cellulose nanocrystals. 2020. **12**(30): p. 34235-34244.

25. Li, G., et al., A stretchable and adhesive ionic conductor based on polyacrylic acid and deep eutectic solvents. npj Flexible Electronics, 2021. **5**(1): p. 23.

26. Kang, J., J.B.H. Tok, and Z. Bao, *Self-healing soft electronics.* Nature Electronics, 2019. **2**(4): p. 144-150.

27. Vatankhah-Varnosfaderani, M., et al., Chameleon-like elastomers with molecularly encoded strain-adaptive stiffening and coloration. Science, 2018. **359**(6383): p. 1509-1513.

28. Reilly, R.B., T.C.J.T. Lee, and H. Care, *Electrograms (ecg, eeg, emg, eog).* 2010. **18**(6): p. 443-458.

29. Cheng, S., et al., Ultrathin Hydrogel Films toward Breathable Skin‐Integrated Electronics. 2023. **35**(1): p. 2206793.

30. Lim, C.Y., et al., Desulfurization performance of choline chloride-based deep eutectic solvents in the presence of graphene oxide. 2020. **7**(11): p. 97.

31. Zhang, W., et al., Skin-like mechanoresponsive self-healing ionic elastomer from supramolecular zwitterionic network. Nature Communications, 2021. **12**(1): p. 4082.

32. Smedley, S.I., *The interpretation of ionic conductivity in liquids*. 2012: Springer Science & Business Media.

33. Lim, C., et al., Tissue-like skin-device interface for wearable bioelectronics by using ultrasoft, mass-permeable, and low-impedance hydrogels. 2021. **7**(19): p. eabd3716.

34. Guimarães, C.F., et al., *The stiffness of living tissues and its implications for tissue engineering.* Nature Reviews Materials, 2020. **5**(5): p. 351-370.

35. Liu, S., et al., *Strategies for body-conformable electronics.* 2022. **5**(4): p. 1104-1136.

36. Ashbaugh, D.R., Quantitative-qualitative friction ridge analysis: an introduction to basic and advanced ridgeology. 1999: CRC press.

37. Hurst, J.W.J.C., Naming of the waves in the ECG, with a brief account of their genesis. 1998. **98**(18): p. 1937-1942.

38. Fujimura, T., M.J.S.R. Hotta, and Technology, The preliminary study of the relationship between facial movements and wrinkle formation. 2012. **18**(2): p. 219-224.

39. Kandel, E.R., et al., *Principles of neural science*. Vol. 4. 2000: McGraw-hill New York.

40. Mari-Acevedo, J., K. Yelvington, and W.O. Tatum, *Chapter 9 - Normal EEG variants*, in *Handbook of Clinical Neurology*, K.H. Levin and P. Chauvel, Editors. 2019, Elsevier. p. 143-160.

41. French, J.A., *Foreword*, in *Rowan's Primer of EEG (Second Edition)*, L.V. Marcuse, M.C. Fields, and J. Yoo, Editors. 2016, Elsevier: London. p. vii.

42. Twitmyer, E.B.J.J.o.E.P., *A study of the knee jerk.* 1974. **103**(6): p. 1047.

43. Horwitz, A.V. and J.C. Wakefield, The loss of sadness: How psychiatry transformed normal sorrow into depressive disorder. 2007: Oxford University Press.

44. Fekadu, N., W. Shibeshi, and E.J.J.D.A. Engidawork, *Major depressive disorder: pathophysiology and clinical management.* 2017. **6**(1): p. 255-257.

45. Baroiu, L., et al., Assessment of Depression in Patients with COVID-19. 2021. **12**(2): p. 254-264.

46. de Aguiar Neto, F.S., J.L.G.J.N. Rosa, and B. Reviews, *Depression biomarkers using non-invasive EEG: A review.* 2019. **105**: p. 83-93.

47. Voetterl, H.T.S., et al., Alpha peak frequency-based Brainmarker-I as a method to stratify to pharmacotherapy and brain stimulation treatments in depression. Nature Mental Health, 2023. **1**(12): p. 1023-1032.

48. Márton, L., et al., *Detrended fluctuation analysis of EEG signals.* 2014. **12**: p. 125-132.

49. Duan, L., et al., Machine learning approaches for MDD detection and emotion decoding using EEG signals. 2020. **14**: p. 284.

50. Yin, L., C. Zhang, and Z.J.C.C. Cui, Experimental research on real-time acquisition and monitoring of wearable EEG based on TGAM module. 2020. **151**: p. 76-85.

51. Wu, Y., N.J.C. Xie, and M.M.i. Medicine, *Attention Optimization Method for EEG via the TGAM.* 2020. **2020**.

52. Acharya, U.R., et al., Computer-aided diagnosis of depression using EEG signals. 2015. **73**(5-6): p. 329-336.

53. Huang, S., et al., Applications of support vector machine (SVM) learning in cancer genomics. 2018. **15**(1): p. 41-51.

54. Cai, H., et al., Feature-level fusion approaches based on multimodal EEG data for depression recognition. 2020. **59**: p. 127-138.

55. Zulfiker, M.S., et al., An in-depth analysis of machine learning approaches to predict depression. 2021. **2**: p. 100044.

56. Cacheda, F., et al., Early detection of depression: social network analysis and random forest techniques. 2019. **21**(6): p. e12554.

57. Menze, B.H., et al., A comparison of random forest and its Gini importance with standard chemometric methods for the feature selection and classification of spectral data. 2009. **10**: p. 1-16.

58. Jamal, R. and M. Kullar, Multivariate Time-Series Signals of Affect Dynamics and Neural Oscillations with Suggested Applications to Technology to Identify Depression Risk. 2022.

59. Roh, S.-C., et al., EEG beta and low gamma power correlates with inattention in patients with major depressive disorder. 2016. **204**: p. 124-130.

60. Watts, D., et al., Predicting treatment response using EEG in major depressive disorder: A machine-learning meta-analysis. 2022. **12**(1): p. 332.

61. Meerwijk, E.L., J.M. Ford, and S.J.J.B.p. Weiss, Resting-state EEG delta power is associated with psychological pain in adults with a history of depression. 2015. **105**: p. 106-114.

62. De Stefani, E. and D.J.F.i.P. De Marco, Language, gesture, and emotional communication: An embodied view of social interaction. 2019. **10**: p. 2063.

63. Qi, J., et al., Computer vision-based hand gesture recognition for human-robot interaction: a review. 2023: p. 1-26.

64. Kim, J., S. Mastnik, and E. André. EMG-based hand gesture recognition for realtime biosignal interfacing. in Proceedings of the 13th international conference on Intelligent user interfaces. 2008.
